# Pachydictyols B and C: New Diterpenes from *Dictyota* *dichotoma* Hudson

**DOI:** 10.3390/md11093109

**Published:** 2013-08-22

**Authors:** Ghada S. E. Abou-El-Wafa, Mohamed Shaaban, Khaled A. Shaaban, Mohamed E. E. El-Naggar, Armin Maier, Heinz H. Fiebig, Hartmut Laatsch

**Affiliations:** 1 Department of Botany, Faculty of Science, Mansoura University, Algomhuria St. 60, El-Mansoura 35516, Egypt; E-Mail: dodymarine99@yahoo.com; 2 Institute of Organic and Biomolecular Chemistry, University of Göttingen, Tammannstrasse 2, D-37077 Göttingen, Germany; E-Mails: kelbarbary100@yahoo.com (K.A.S.); hlaatsc@gwdg.de (H.L.); 3 Chemistry of Natural Compounds Department, Division of Pharmaceutical Industries, National Research Centre, El-Behoos St. 33, Dokki-Cairo 12622, Egypt; 4 Oncotest GmbH, Am Flughafen 12-14, D-79108 Freiburg, Germany; E-Mails: armin.maier@oncotest.de (A.M.); heiner.fiebig@oncotest.de (H.H.F.)

**Keywords:** marine brown alga, *Dictyota dichotoma*, pachydictyols, diterpenes, bioactivity

## Abstract

Two new diterpenoids, pachydictyol B (**1a**/**1b**) and C (**2**), were isolated from the dichloromethane extract of the marine brown alga, *Dictyota dichotoma*, collected from the Red Sea coast of Egypt, along with the known metabolites, pachydictyol A (**3a**), dictyol E (**4**), *cis*-africanan-1α-ol (**5a**), fucosterol (**6**), tetrahydrothiophen-1,1-dioxide and poly-β-hydroxybutyric acid. GC-MS analysis of the nonpolar fractions also indicated the presence of β-bourbonene and nonanal, along with three hydrocarbons and five fatty acids or their simple derivatives, respectively. GC-MS analysis of the unsaponifiable algal petroleum ether extract revealed the presence of a further eight compounds, among them 2,2,6,7-tetramethyl-10-oxatricyclo[4.3.0.1(1,7)]decan-5-one (**7**), *N*-(4-bromo-*n*-butyl)-piperidin-2-one (**8**) and *tert*-hexadecanethiol. Structures **1**–**6** were assigned by 1D and 2D NMR, mass spectra (EI, CI, HREI and HRESI) and by comparison with data from related structures. The crude algal extract was potently active against the breast carcinoma tumor cell line, MCF7 (IC_50_ = 0.6 µg mL^−1^); pachydictyol B (**1a**) and dictyol E (**4**) showed weak antimicrobial properties, and the other compounds were inactive. Pachydictyols B (**1a**) and C (**2**) demonstrated a weak and unselective cytotoxicity against twelve human tumor cell lines with a mean IC_50_ of >30.0 µM.

## 1. Introduction

Brown algae belonging to the family, *Dictyotaceae*, are a rich source of biologically active isoprenoids [1,2]. About 200 diterpenoids, belonging to 15 chemical classes, have been isolated from *Dictyota* spp. [3,4,5]. Some of these compounds are reported to display significant cytotoxic, antiviral, feeding-deterrent and antifouling activities [3,6-10] or were useful for chemotaxonomic and biogenic studies of the genus, *Dictyota* [11,12]. The production of secondary metabolites in other genera of benthic marine brown algae has also been reported and is often associated with protection against herbivores [13].

**Figure 1:**
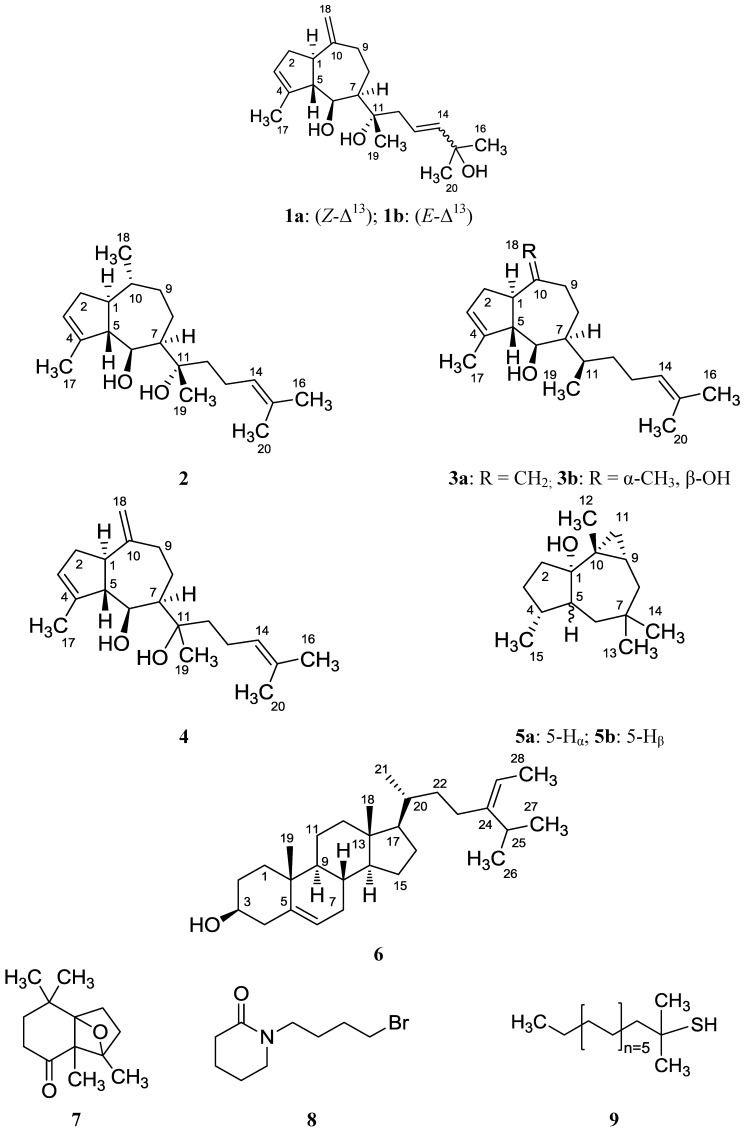
Structures of compounds **1**–**9**.

During our search for bioactive diterpenoids from marine sources, the brown alga, *Dictyota dichotoma* (Hudson) Lamouroux, from the Red Sea, was selected for further investigation on the basis of notable *in vitro* cytotoxicity of a crude extract against the breast carcinoma tumor cell line, MCF7 (IC_50_ = 0.6 µg mL^−1^) and on the basis of chemical screening by TLC. Several UV-inactive bands ranging from low to high polarity were detected that turned pink or gave a blue-violet color after spraying with anisaldehyde/sulfuric acid, suggesting the presence of isoprenoids. Soxhlet extraction of the algae using dichloromethane, followed by a series of chromatographic steps, afforded three new diterpenes, *cis*- and *trans*-pachydictyol B (**1a**/**1b**) and pachydictyol C (**2**), see Figure 1. Additionally, the known metabolites, pachydictyol A (**3a**) [3], dictyol E (**4**) [14,15], *cis*-africanan-1α-ol (**5a**) [16], fucosterol (**6**), poly-β-hydroxybutyric acid and tetrahydrothiophene-1,1-dioxide, were isolated. GC-MS analyses of the nonpolar fraction and of the unsaponifiable residue of the algal extract revealed 18 further components, among them **7**–**9** (Supplementary Material, Tables S1 and S2).

## 2. Results and Discussion

### 2.1. Structure Analysis and Characterization of Isolated Compounds

Separation of *D. dichotoma* extracts on silica gel delivered eight compounds with a wide range of polarities. Under TLC monitoring, four compounds of moderate to high polarity were especially conspicuous. They were not UV absorbing, but stained intensely violet when sprayed with anisaldehyde/sulfuric acid. The least polar compound and a moderately polar component were identified as pachydictyol A (**3a**) [3] and dictyol E (**4**) [14,15], respectively, by means of NMR and MS data. The other two compounds showed a close similarity to **3a** and **4** and appeared to be new derivatives thereof.

Compound **1a** was obtained as polar colorless oil, with a molecular weight of *m/z* 320 Dalton by DCI MS. EI MS showed two characteristic fragment ions resulting from the successive loss of two molecules of water. (+)-HRESI MS confirmed the molecular formula as C_20_H_32_O_3_, with the same number of double bond equivalents as in **3a**, but with two more oxygen atoms.

The ^13^C NMR/HMQC spectra of **1a** confirmed the expected twenty carbon signals and pointed to a close structural similarity with **3a** and **4**. The olefinic carbons of **1a** had nearly the same shifts as for **3a**/**4**; however, they were assigned by HSQC to three olefinic methines, two sp^2^ C_q_ atoms and one exocyclic methylene group (3/2/1) instead of 2/3/1, as in **3a**/**4**. Between δ_C_ 70~76, there were three signals from oxygenated carbons visible in the spectrum of **1a**, but only one for **3a** and two for **4**, respectively. This indicated a new dihydroxypachydictyol A, that we named pachydictyol B (**1a**).

An intensive spectroscopic study of compound **1a** revealed the same octahydroazulen-4-ol parent structure substituted at the 7-position as found in pachydictyol A (**3a**) and dictyol E (**4**) (Table 1, Table 2). The sp^2^ methylene protons H_2_-18 displayed HMBC correlations with the quaternary carbon C-10 (δ_C_ 151.6, ^2^*J*) and its neighboring methine CH-1 (δ_C_ 46.0, ^3^*J*) and methylene CH_2_-9 (δ_C_ 40.3) carbons. The angular methine proton at C-1 (δ_H_ 2.50) showed three HMBC correlations, with the methine carbon CH-5 (δ_C_ 59.8, ^2^*J*), the oxy-methine CH-6 (δ_C_ 73.9, ^3^*J*) and the CH_2_-9 signal (δ_C_ 40.3, ^3^*J*), respectively.

**Table 1:** ^13^C and ^1^H NMR data of pachydictyols A (**3a**) and B (**1a**/**1b**) in CDCl_3_ (*J* in [Hz]).

position	*cis*-Pachydictyol B (1a)		*trans*-Pachydictyol B (1b)		Pachydictyol A (3a)	
	δ_C_^(a)^	δ_H_^(b)^	δ_C_^(a)^	δ_H_^(c)^	δ_C_^(a)^	δ_H_^(b)^
1	46.0	2.50 (m)	46.1	2.52 (m)	46.1	2.67 (m)
2	33.6	2.43 (m), 2.13 (m)	33.7	2.46 (m), 2.16 (m)	33.9	2.50 (m), 2.22 (m)
3	123.9	5.28 (m)	124.2	5.30 (m)	123.9	5.33 (m)
4	140.8	-	140.7	-	141.3	-
5	59.8	2.33 (m)	59.9	2.36 (m)	60.4	2.30 (m)
6	73.9	4.18 (dm, 7.6)	74.1	4.18 (m)	75.1	3.92 (d, 7.8)
7	48.6	1.56 (m)	49.0	1.58 (m)	47.8	1.55 (m)
8	21.6	1.69 (m)	21.6	1.73 (m), 1.65 (m)	23.6	1.50 (m)
9	40.3	2.60 (m), 2.04 (m)	40.3	2.62 (dm, 15.7 Hz), 2.06 (m)	40.6	2.62 (m), 2.10 (m)
10	151.6	-	151.5	-	152.4	-
11	75.9	-	76.0	-	34.8	1.21 (m)
12	43.8	2.42 (m), 2.33 (m)	44.0	2.47 (m), 2.37 (m)	35.1	2.25 (m), 1.53 (m)
13	122.1	5.63 (br m)	126.4	5.68 (dt, 15.6, 8.0)	25.7	2.04 (m), 1.95 (m)
14	141.6	5.64 (br m)	137.4	5.60 (d, 15.6)	124.6	5.13 (tq, 8.6, 1.3)
15	70.4	-	81.6	-	131.4	-
16	29.4	1.24 (s)	24.7	1.25 (s)	25.8	1.68 (s)
17	15.8	1.77 (s)	15.8	1.77 (s)	15.9	1.81 (d, 1.3)
18	107.3	4.72 (br s), 4.69 (br s)	107.4	4.74 (s), 4.70 (s)	107.0	4.74 (br s)
19	25.4	1.15 (s)	25.5	1.17 (s)	17.6	0.99 (d, 6.0)
20	29.8	1.25 (s)	24.0	1.28 (s)	17.7	1.61 (s)

^(a)^ 125 MHz; ^(b)^ 300 MHz; ^(c)^ 600 MHz.

**Table 2:** ^13^C and ^1^H NMR data of pachydictyol C (**2**), dictyol C (**3b**) and dictyol E (**4**) in CDCl_3_ (*J* in [Hz]).

position	Pachydictyol C (2)		Dictyol C (3b) [14]		Dictyol E (4)	
	δ_C_^(a)^	δ_H _	δ_C_^(a)^	δ_H_^(b)^	δ_C_^(a)^	δ_H _
1	49.1	1.25 (m)	49.1	2.21	46.1	2.53 (q, 9.8)
2	33.0	2.21 (m)	32.9	n.r.	33.7	2.44 (m), 2.16 (m)
3	123.2	5.26 (br m)	123.4	5.26 (br s)	123.9	5.28 (br m)
4	142.4	-	142.5	-	140.8	-
5	52.7	2.75 (m)	52.7	2.74 (dd,7.8, 6.0)	60.0	2.34 (m)
6	74.5	3.86 (dd, 8.2, 3.4)	74.4	3.87 (dd,7.8, 3.6)	74.1	4.14 (dd, 7.9, 2.7)
7	50.0	2.15 (m)	49.9	n.r.	48.3	1.60 (m)
8	19.8	1.27 (m), 1.22 (m)	19.7	n.r.	21.5	1.71, 1.61 (2 m)
9	34.5	1.51 (m)	46.6	n.r.	40.4	2.63 (dm, 14.5), 2.06 (m)
10	34.9	1.19 (m)	72.4	-	151.7	-
11	72.6	-	34.4	n.r.	76.1	-
12	46.6	1.40 (m), 1.88 (m)	34.7	n.r.	40.9	1.67 (m)
13	25.6	2.02 (m), 1.94 (m)	25.5	n.r.	23.2	1.99 (m)
14	124.7	5.14 (m)	124.7	5.14 (br t, 7.1)	124.2	5.10 (t, 7.1)
15	131.3	-	131.6	-	131.3	-
16	25.8	1.68 (s)	25.7	1.62 (d, 0.9)	25.6	1.64 (s)
17	16.3	1.82 (s)	16.3	1.85 (dd, 2.0, 1.2)	15.7	1.77 (s)
18	17.5	0.97 (d, 6.4)	30.0	1.22 (s)	107.3	4.73 (s), 4.70 (br d, 1.3)
19	30.0	1.19 (s)	17.5	1.00 (d, 6.6)	25.1	1.18 (s)
20	17.7	1.60 (s)	17.7	1.70 (s)	17.6	1.57 (s)

^(a)^ 125 MHz; ^(b)^ 300 MHz; n.r., signals not reported.

**Figure 2:**
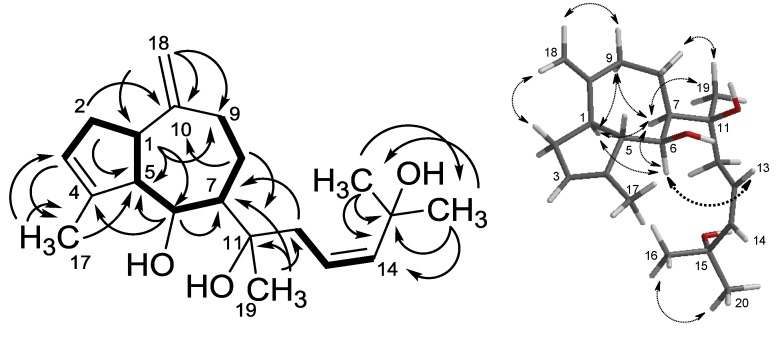
Left: H,H COSY (▬) and selected HMBC (↔) correlations in *cis*-pachydictyol B (**1a**); right: selected NOESY couplings in a preferred conformation of **1a**.

COSY and HMBC correlations were seen from CH_2_-2 (δ_H_ 2.43, 2.13) to the olefinic methine CH-3 (δ_H_ 5.28) and to CH-1 (δ_C_ 46.0). A ^3^*J*_CH_ coupling from the olefinic methyl CH_3_-17 (δ_H_ 1.77) to CH-3 (δ_C_ 123.9), to C_q_-4 (δ_C_ 140.8) and CH-5 (δ_C_ 59.8) completed the methyl-cyclopentene partial structure. The remaining two carbons of the octahydroazulene, CH-7 (δ_C_ 48.6, δ_H_ 1.56) and CH_2_-8 (δ_C_ 21.6, δ_H_ 1.69), were assigned through contiguous H,H COSY correlations between CH_2_-9 (δ_H_ 2.60, 2.04), CH_2_-8 (δ_H_ 1.69), CH-7 (δ_H_ 1.56) and CH-6 (δ_H_ 4.18) and confirmed by H→C (HMBC) correlations (see Figure 2, left).

A 1,2-disubstituted ethanediyl (–CH=CH–), *gem*-dimethyls bound to a quaternary oxycarbon [(CH_3_)_2_C_q_(OH)–] and a –C_q_(OH,CH_3_)–CH_2_– fragment were identified as sub-structures of the side chain C_8_H_15_O_2_ and connected by HMBC correlations (see Figure 2), resulting in the planar structure, **1a**/**1b**. The high similarity of ^1^H and ^13^C NMR shifts of the chiral centers in **1a** and **4** (Table 1), as well as NOESY correlations (Figure 2, right), indicated the same relative configuration as found in pachydictyol A (**3a**) and dictyol E (**4**): proton H-1 gave NOE signals with H-6 and H-7 and H-7 coupled with H-6 and H-9α (δ 2.04), and H-1 gave a cross signal with H-2α (δ 2.13), indicating a *syn*-facial orientation of all these hydrogens. This assignment was supported by strong NOE signals between δ 2.43 (H-2β) and δ 4.69 (*Z*-H-18) or δ 2.60 (H-9β) and δ 4.72 (*E*-H18), respectively. This agrees very well with *semi-*empirical calculations [17]. These indicated that in the energy minimum, the exocyclic double bond and the C2_β_ and C9_β_ hydrogens are nearly in the same plane and much closer to CH_2_-18 than the respective α-protons. The relative configuration in the ring system of **1a**/**1b**, **3a** and **4** is, therefore, certainly the same, and for biosynthetic reasons, the same absolute configuration can also be assumed.

The configuration at C-11 was estimated tentatively on the basis of the expected dominating conformation. By AM1 [17], about 5500 conformers were calculated using the Monte Carlo method, of which 99.3% in the Boltzmann distribution (19 of 22 molecules in a range of ~15 kJ/mol above the global minimum) all showed a hydrogen bridge between the hydroxy groups, 6-OH and 11-OH. Due to the restricted rotation around the C-7/C-11 bond resulting thereby, the (11*S*) configuration with the 11-methyl in β-orientation and an 11α chain (C12-C16) or the corresponding (11*R*) diastereomer might be differentiated by NOESY data. On this basis, the strong NOE between the double bond protons H-13/14 and both H-6 and H-7 was taken as a clear indication of the (11*S*) configuration.

In deuteriochloroform at 300 MHz, a ^3^*J* coupling between H-13/14 was not visible, due to nearly identical shifts. Inspection of further **1a** fractions revealed, however, a second isomer with slightly different ^1^H and ^13^C shifts in the region of C-13–C-16/20. In this compound, the olefinic proton, H-14, appeared as a doublet (*J* = 15.9 Hz), while H-13 gave a doublet of a triplet (*J* = 15.9, 6.4 Hz), clearly indicating an (*E*)-configuration of the side chain (Supplementary Material, Figure S7). As all 2D correlations of both isomers, along with the shifts of the chiral centers, were identical (Figure 2 and Figure S11, Supplementary Material), **1a** and **1b** were determined to be *cis*- and *trans*-pachydictyol B, respectively.

**Figure 3:**
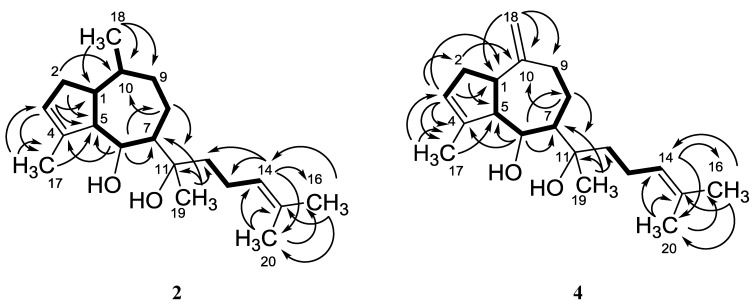
H,H COSY (▬) and selected HMBC (→) couplings in pachydictyol C (**2**) and dictyol E (**4**).

A further diterpene **2** was also obtained as a colorless oil. It had similar chromatographic properties as **1a**/**1b**, but a slightly lower polarity. (+)-HRESI MS established the molecular formula as C_20_H_34_O_2_, and EI MS delivered fragment ions at *m/z* 288 and 270, again due to the successive elimination of two water molecules. In the ^1^H NMR spectrum, compound **2** displayed the same pattern as dictyol E (**4**), except that the two exocyclic sp^2^-methylene signals of CH_2_-18 in **4** were replaced in **2** by a methyl doublet at δ_H_ 0.97 (*J* *=* 6.4 Hz), with the coupling partner, H-10, giving a multiplet at δ_H_ 1.19. All other shifts and coupling patterns were similar to those of dictyol E (**4**) (Table 2). The ^13^C NMR data were identical, within the limits of error, to those of dictyol C (**3b**) [14], with the exception of the shifts for C-11/12 and C-18/19 in **2** that were pairwise exchanged against C-10/9 and C-19/18 in **3b** (see Table 2 and Figure 3). Accordingly, **2** was confirmed as 5-(1-hydroxy-1,5-dimethylhex-4-enyl)-3,8-dimethyl-1,3a,4,5,6,7,8,8a-octahydro-azulen-4-ol and named pachydictyol C.

**Figure 4:**
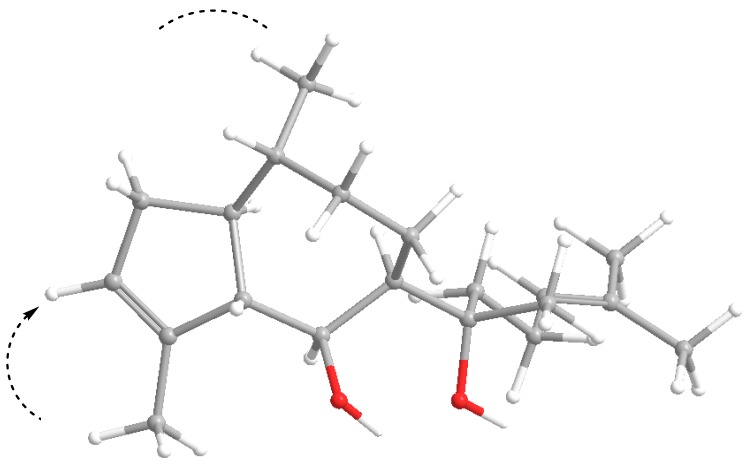
Selected NOESY couplings of pachydictyol C (**2**).

In contrast to **1a**, **3a** and **4**, compound **2** showed a negative optical rotation ([α]^20^_D_ = −15°), similar to that of the related 10-methyl derivative dictyol C (**3b**) ([α]^20^_D_ = −16.6°) [14]. The configuration of **2** was assumed to be the same as in **1**, **3a** and **4**, with regard to the common biosynthetic origin of all dictyols isolated here, because of the closely related shifts of the respective atoms in **3b** and **4** and on the basis of similar NOESY correlations (Figure 4). As H-6 showed a strong NOESY correlation with H-1 and a weaker one with CH_3_-18, an α-orientation with an equatorial position of this methyl must be assumed. This is confirmed by a clear correlation of H-10 with CH_2_-13 and CH-14, which both must be placed on the β-face, resulting in a (10*R*) configuration. Consequently, this compound was assigned as (1*S*,5*S*,6*S*,7*R*,10*R*,11*S*)-**2**.

**Figure 5:**
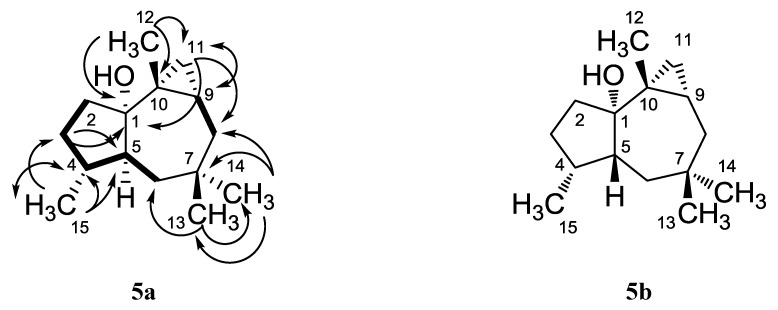
H,H COSY (▬, ↔) and selected HMBC (→) couplings of *cis*-africanan-1α-ol (**5a**), along with the structure of *trans*-africanan-1α-ol (**5b**).

Compound **5a** did not absorb UV light as well as the other compounds isolated and gave a pink color with anisaldehyde/sulfuric acid. It was obtained as a nonpolar colorless oil with the molecular formula, C_15_H_26_O, and identified as *cis*-africanan-1α-ol, whose structure **5a** had been reported previously [16], but was not completely characterized. We report herein the first full NMR assignment of **5a**, based on 2D experiments (Table 3, Figure 5).

**Table 3:** ^13^C and ^1^H NMR data of *cis*-africanan-1α-ol (**5a**) in CDCl_3_ (*J* in [Hz]).

Position	*cis*-african-1α-ol (5a) Isolated		*cis*-africanan-1α-ol (5a) [16]		*trans-*africanan-1α-ol (5b) [18]	
	δ_C_^(a)^	δ_H_^(b)^	δ_C_	δ_H_	δ_C_	δ_H_
1	83.2	-	85.3	-	85.9	-
2	41.3	1.97 (m), 1.52 (m)	38.9*	1.47 (m), 1.97 (m)	38.1	1.88 (m), 1.92 (m)
3	32.7	1.67 (m), 1.38 (m)	32.7	1.68 (m), 1.35 (m)	30.1	1.96 (m), 1.17 (m)
4	43.3	1.32 (m)	43.2	1.31 (m)	38.1	1.74 (m)
5	55.0	1.20 (m)	54.9	1.09 (m)	49.5	1.05 (ddd, 11.7, 10.5, 2.7)
6	41.8	1.06 (m), 1.00 (m)	41.7	0.99 (m), 1.38 (m)	39.8	1.19 (ddd, 14.4, 2.7, 2.1), 1.28 (dd, 14.4, 11.7)
7	33.3	-	33.3	-	33.0	-
8	38.9	1.04 (m), 1.49 (m)	41.2 *	1.05, 1.47	39.7	1.89 (dd, 15.0, 11.8), 1.73 (ddd, 15.0, 5.5, 2.1)
9	22.3	0.81 (m)	22.2	0.79 (m)	25.7	0.74 (m)
10	23.6	-	23.5	-	26.9	-
11	15.3	0.66 (dd, 6.4, 5.2), 0.28 (dd, 8.6, 4.1)	15.2	0.66 (m), 0.27 (m)	16.3	0.74 (m), 0.31 (m)
12	26.8 *	1.03 * (s)	18.9	1.03 (s)	23.5	1.12 (s)
13	28.3 *	0.84 * (s)	29.1	0.98 (s)	35.1	0.96 (s)
14	29.2 *	0.98 * (s)	28.2	0.84 (s)	28.0	0.94 (s)
15	18.9 *	1.02 * (d, 6.5)	26.7	1.02 (d)	19.7	0.93 (d, 6.5)

^(a)^ 125 MHz; ^(b)^ 300 MHz; * differently assigned in the literature.

In addition, tetrahydrothiophene-1,1-dioxide (sulfolan) was isolated from the nonpolar fraction I [19], while fucosterol (**6**) and poly-β-hydroxybutyric acid [20,21] were isolated from fraction II. Their structures were confirmed by comparison of their spectroscopic data with that in the literature. Subsequent GC-MS analysis of fraction I and of the unsaponifiable part of a petroleum ether extract of *D. dichotoma* revealed a further ten (Supplementary Material, Tables S1) and eight compounds (Supplementary Material, Tables S2), respectively, among them 2,2,6,7-tetramethyl-10-oxatricyclo­ [4.3.0.1(1,7)]decan-5-one (**7**), *N*-(4-bromo-*n*-butyl)-piperidin-2-one (**8**) and *tert*-hexadecanethiol (**9**).

### 2.2. Biological Activities

The crude algal extract showed notable *in vitro* cytotoxicity against the breast carcinoma tumor cell line, MCF7 (IC_50_ = 0.6 µg mL^−1^), but showed only marginal cytotoxicity against brine shrimp (3.1% at 100 µg mL^−1^) [22,23]. In the agar diffusion test, extracts of *D. dichotoma* were not active against bacteria (*Bacillus subtilis*, *Staphylococcus aureus*, *Streptomyces viridochromogenes* (Tü 57), *Escherichia coli*), fungi (*Candida albicans*, *Mucor miehei*, *Rhizoctonia solani* and *Pythium ultimum*) [24] or the microalgae, *Chlorella vulgaris*, *C. sorokiniana* and *Scenedesmus subspicatus*, as a test for phytotoxicity, at concentrations of 100 µg/disc [25].

(*Z*)-Pachydictyol B (**1a**) displayed high antimicrobial activity in the agar diffusion test at 10 µg/paper disc against *Mucor miehei* (20 mm) and was weakly active against *Candida albicans* (11 mm) and *Pythium ultimum* (12 mm). Pachydictyol C (**2**) showed no antimicrobial activity, and both **1a** and **2** were not toxic towards brine shrimp at 10 µg mL^−1^. At the time of isolation of pure compounds, the MCF7 test was no longer available and has been substituted by other cell lines (Table 4); the *in vitro* examination demonstrated weak and unselective cytotoxicity against twelve human tumor cell lines, with a mean IC_50_ of >30.0 µg mL^−1^; the high activity of the crude extract could not be reproduced.

**Table 4:** Cytotoxic activities of pachydictyols A–C (**3a**, **1a**, **2**), Dictyol E (**4**), *cis*-africanan-1α-ol (**5a**) and fucosterol (**6**).

Compound	Antitumor Potency ^a^		Tumor Selectivity ^b^	
	Mean IC_50_ (µM)	Mean IC_70_ (µM)	*n*/total	%
*cis*-pachydictyol B (**1a**)	>30.0	>30.0	0/12	0
pachydictyol C (**2**)	>30.0	>30.0	0/12	0
pachydictyol A (**3a**)	23.6	>30.0	0/12	0
dictyol E (**4**)	>30.0	>30.0	0/12	0
*cis*-africanan-1α-ol (**5a**)	>10.0	>10.0	0/12	0
fucosterol (**6**)	19.5	>30.0	0/12	0

^a^ Mean IC_50/70_ values, determined as the average of 12 human tumor cell lines tested. Individual IC_50_ < ½ mean IC_50_; e.g., if the mean IC_50_ = 2.0 µM, the threshold for the above average sensitivity was IC_50_ < 1.0 µM; ^b^ the tumor cell lines are: BXF, bladder; CEXF, cervix; CX,F colorectal; GXF, gastric; LXF, lung; MAXF, breast; MEXF, melanoma xenograft; OVXF, ovarian cancer xenograft; PRXF, prostate; PXF, pleuramesotheliom; RXF, renal; and UXF, uterus body, with XF = Xenograft Freiburg-derived cell line; A, adeno; L, large cell; E, epidermoid cell; S, small cell.

## 3. Experimental Section

### 3.1. General Experimental Procedures

NMR shifts were referenced on the solvent signal of CDCl_3_ (δ_H_ = 7.27, δ_C_ = 77.0; 300 or 600 MHz for ^1^H and 125 Hz for ^13^C). GC-MS spectra were measured on a Trace GC-MS Thermo Finnigan chromatograph, using EI ionization mode (70 eV) and a CP-Sil 8 CB capillary column for amines (length: 30 m; inside diameter: 0.25 mm; outside diameter: 0.35 mm; film thickness: 0.25 µm). The analysis was carried out using a temperature program. The initial temperature was 40 °C (maintained for 1 min), and the temperature was then ramped up at a rate of 10 °C/min to a final temperature of 280 °C (kept for 10 min). The injector and detector temperature were 250 °C, and He was used as the carrier gas at a flow rate of 1 mL min^−1^. The total run time was 27 min, and the injection volume was 0.2 µL. For details see reference [26].

### 3.2. Collection and Taxonomy of the Marine Alga

The brown alga, *Dictyota dichotoma* (Huds) Lamour, was collected in the summer of 2007 at Ras Abu-Bakr, 65 km north of Ras Gharib on Suez-Gulf, Red Sea, Egypt. The identification was carried out by Abou-ElWafa according to Nasr’s method [27,28]. A reference specimen of the alga is kept at the Department of Botany, Faculty of Science, Mansoura University, Egypt.

Samples of *Dictyota dichotoma* (Huds) Lamour were separated from epiphytes and the dead matrix in running water and rinsed several times in distilled water. The sample was then spread on string nets, allowed to dry in air, ground and stored in closed bottles at room temperature.

### 3.3. Extraction and Isolation of the Bioactive Constituents

The air-dried algal material (~360 g) was extracted in a Soxhlet apparatus for ~12 h using dichloromethane (DCM). The DCM extract was filtered and the solvent evaporated *in vacuo* at 40 °C, affording 14.3 g of a greenish brown crude extract. This extract was fractionated on a silica gel column, eluting with petroleum ether (boiling range 40–60 °C)-DCM and DCM-MeOH gradients, delivering five fractions: I (0.11 g), II (3.2 g), III (2.3 g), IV (2.6 g) and V (5.1 g). TLC monitoring was used, with anisaldehyde/sulfuric acid as the spraying reagent. The first nonpolar fraction I was submitted to GC-MS analysis, detecting the existence of tetrahydrothiophen-1,1-dioxide, β-bourbonene and nine further compounds (Supplementary Material, Tables S1). A preparative separation of fraction I on silica gel (eluting with a cyclohexane-DCM gradient) afforded a pale yellow oil, which was further purified on Sephadex LH-20 (DCM/40% MeOH) to give the colorless, oily, tetrahydrothiophene-1,1-dioxide (9 mg, 0.06%). Fraction II was applied to a Sephadex LH-20 (DCM/40% MeOH) to afford two sub-fractions, IIa (0.7 g) and IIb (2.4 g). Sub-fraction IIa was not further investigated. Sub-fraction IIb (2.4 g) was washed with methanol to give the insoluble, colorless solid, poly-β-hydroxybutyric acid (1.47 g, 10.3%). The soluble part of the methanolic extract (0.88 g) was applied to a silica gel column and eluted with a cyclohexane/DCM gradient to deliver pachydictyol A (**3a**) (18.2 mg, 0.12%) and *cis*-africanan-1α-ol (**5a**) (13.1 mg, 0.09%) as colorless oils. Further purification of sub-fraction IIb afforded fucosterol (**6**, 30.2 mg, 0.21%) as a colorless solid. The eluates from fractions III and IV were combined (4.9 g) and purified on a Sephadex LH-20 column (MeOH) to give sub-fraction IIIa (2.5 g). The latter was further purified by silica gel column chromatography, eluting with DCM-MeOH gradients, to afford the colorless oily compound, dictyol E (**4**, 55.0 mg, 0.38%). The last polar fraction V was separated by column chromatography on silica gel, again eluting with DCM-MeOH gradients, to give sub-fractions Va (1.2 g) and Vb (50.2 mg). Sub-fraction Va was purified by PTLC (DCM) and a subsequent silica gel column (cyclohexane-DCM) to yield pachydictyol C (**2**, 8.0 mg, 0.06%) as a colorless oil. Finally, purification of sub-fraction Vb on silica gel (DCM-MeOH) afforded (*Z*)-pachydictyol B (**1a**, 30.0 mg, 0.21%) and (*E*)-pachydictyol B (**1b**, 13 mg, 0.09%) as colorless oils.

Tetrahydrothiophene-1,1-dioxide: Colorless oil, UV non-absorbing, turned brown on spraying with anisaldehyde/sulfuric acid or by PdCl_2_ (0.5% in water) and heating; *R*_f_ = 0.68 (CH_2_Cl_2_/5% MeOH); ^1^H NMR data (300 MHz, in CDCl_3_): δ = 3.05 (m, 4H), 2.22 (m, 4H); ^13^C NMR data (75 MHz, in CDCl_3_): δ = 50.9 (2 CH_2_), 22.5 (2 CH_2_); EI-MS (70 eV): *m/z* (%) = 122 (^34^S[M]^+•^, 2), 120 (^32^S[M]^+•^, 44), 56 ([M − SO_2_]^+•^, 96), 55 ([M − HSO_2_]^+^, 72), 48 (6), 41 (100); HREI-MS: *m/z* = 120.0245 (calcd. 120.0245 for C_4_H_8_O_2_S).

*cis*-Pachydictyol B (**1a**): Colorless oil, UV non-absorbing, turned dark violet on spraying with anisaldehyde/sulfuric acid and heating; *R*_f_ = 0.40 (CH_2_Cl_2_/3% MeOH), 0.35 (cyclohexane/50% CH_2_Cl); [α]^20^_D_ +7 (*c* = 0.1, MeOH); ^1^H NMR (300 MHz, in CDCl_3_) and ^13^C NMR (150 MHz, in CDCl_3_), see Table 1; EI-MS (70 eV): *m/z* (%) = 302 ([M − H_2_O]^+•^, 6), 284 ([M − 2H_2_O]^+•^, 12), 241 (4), 221 (22), 203 (14), 175 (10), 159 (48), 145 (35), 133 (24), 107 (20), 105 (22), 82 (62), 71 (18), 55 (14), 43 (100), 41 (25); (+)-DCI-MS: *m/z* (%) = 338 ([M + NH_4_]^+^, 100), 320 ([M + NH_4_ − H_2_O]^+^, 76); (+)-HRESI-MS: *m/z* = 343.22437 [M + Na]^+^ (calcd. 343.22436 for C_20_H_32_O_3_Na).

*trans*-Pachydictyol B (**1b**): The *trans* isomer was obtained as a colorless oil with similar chromatographic properties and mass spectra as found for **1a**, but with a slightly lower polarity (*R*_f_ = 0.45 (CH_2_Cl_2_/3% MeOH); NMR data, see Table 1.

Pachydictyol C (**2**): Colorless oil, UV non-absorbing, turned dark violet on spraying with anisaldehyde/sulfuric acid and heating; *R*_f_ = 0.55 (cyclohexane/50% CH_2_Cl_2_); [α]^20^_D_ −15 (*c* = 0.1, MeOH); ^1^H NMR (300 MHz, in CDCl_3_), ^13^C NMR (150 MHz, in CDCl_3_) see Table 2; EI-MS (70 eV): *m/z* (%) = 306 ([M]^+^, 8), 288 ([M − H_2_O]^+•^, 84), 270 ([M − 2H_2_O]^+•^, 10), 245 (6), 213 (8), 203 (18), 185 (24), 177 (52), 159 (64), 133 (26), 121 (39), 119 (56), 93 (30), 81 (49), 69 (78), 55 (67), 43 (100); (+)-HRESI-MS: *m/z* = 329.24510 [M + Na]^+^ (calcd. 329.24510 for C_20_H_34_O_2_Na).

*cis*-Africanan-1-α-ol (**5a**): Colorless oil, UV non-absorbing, turned pink on spraying with anisaldehyde/sulfuric acid and heating; *R*_f_ = 0.88 (cyclohexane/50% CH_2_Cl_2_); [α]^20^_D_ +7 (*c* = 0.2, MeOH); ^1^H NMR (300 MHz, in CDCl_3_), ^13^C NMR (150 MHz, in CDCl_3_) see Table 3; EI-MS (70 eV): *m/z* (%) = 222 ([M]^+^, 8), 207 ([M − CH_3_]^+^, 16), 175 (12), 159 (34), 125 (58), 95 (38), 81 (46), 69 (88), 41 (100); DCI-MS: *m/z* (%) = 222 ([M + NH_4_ − H_2_O]^+^, 25), 205 ([M − H_2_O]^+^, 100); (+)-HRESI-MS: *m/z* = 245.18773 [M + Na]^+^ (calcd. 245.18766 for C_15_H_26_ONa).

### 3.4. Estimation of Phytosterols and Hydrocarbons

A powdered sample (10 g) of *Dictyota dichotoma* was extracted with petroleum ether (60–80 °C) at room temperature and concentrated *in vacuo* to give an oily residue (70 mg). This extract was then treated with 50 mL of 10% alcoholic KOH and refluxed in a water bath for 2 h. After cooling, 50 mL of water was added, and the solution was extracted with chloroform. The organic phase was washed with water until it became alkali free and was then dried over anhydrous Na_2_SO_4_. The solvent was evaporated to give the unsaponified fraction as oil, which was subsequently subjected to GC-MS analysis [29] (Supplementary Material, Table S2).

### 3.5. Biological Activity Study

Antimicrobial activity was determined according to Burkholder *et al*. [24]. The Brine Shrimp Microwell Cytotoxic Assay was performed according to Takahashi *et al*. and Sajid *et al*. [22,23]. The *in vitro* cytotoxicity test was carried out using the sulforhodamine B SRB assay according to Skehan *et al*. [30].

The antitumor activity testing was performed as follows: A modified propidium iodide assay was used to examine the antiproliferative activity of the compounds against human tumor cell lines. The test procedure has been described elsewhere [31]. Cell lines tested were derived from patient tumors engrafted as a subcutaneously growing tumor in NMRI nu/nu mice or obtained from American Type Culture Collection, Rockville, MD, National Cancer Institute, Bethesda, MD, or Deutsche Sammlung von Mikroorganismen und Zellkulturen, Braunschweig, Germany.

## 4. Conclusions

Three new pachydictyols, namely (*Z*)- and (*E*)-pachydictyols B (**1a**/**1b**) and C (**2**), along with the known pachydictyol A (**3a**), dictyol E (**4**), *cis*-africanan-1α-ol (**5a**), fucosterol (**6**), tetrahydrothiophene-1,1-dioxide and poly-β-hydroxybutyric acid, were isolated from the marine brown alga, *Dictyota dichotoma*. GC-MS analysis of the nonpolar fractions of the algal extract revealed the presence of ten further compounds, whilst the same analysis of the unsaponified petroleum ether extract of the algae detected a further eight compounds (Supplementary Material, Tables S1 and S2). The chemical structures of compounds **1**–**6** were assigned by 1D and 2D NMR spectroscopy, mass spectrometry (EI, CI, HREI, HRESI) and by comparison of the data with that of related structures. The algal extract exhibited no antimicrobial activity against a diverse range of microorganisms and no cytotoxicity against brine shrimp. In contrast to the high anticancer activity of the crude extract against the breast carcinoma tumor cell line, MCF7 (IC_50_ = 0.6 µg mL^−1^), the purified components were only weakly active (Table 4).

## Abbreviations

AM1: Austin Model 1 (a model used in quantum physics)
CI MS: Chemical Ionization Mass Spectra/Mass Spectrometry
COSY: Correlation Spectroscopy
DCI MS: Desorption Chemical Ionization
EI MS: Electron Impact Mass Spectra/Mass Spectrometry
GC-MS analysis: Gas Chromatographic-Mass Spectrometric analysis
HMBC: Heteronuclear Multiple-Bond Correlation
HMQC: Heteronuclear Multiple-Quantum Correlation
HREI MS: High Resolution Electron Impact Mass Spectra/Mass Spectrometry
HRESI MS: High Resolution Electrospray Mass Spectra/Mass Spectrometry
HSQC: Heteronuclear Single Quantum Correlation
NMR: Nuclear Magnetic Resonance
NOE: Nuclear Overhauser Effect
NOESY: Nuclear Overhauser Effect Spectroscopy
PTLC: Preparative Thin-layer Chromatography
TLC: Thin Layer Chromatography

## Supplementary Files

Supplementary File 1 Supplementary Information (PDF, 4646 KB)
