# Supplementary material for: Pachydictyols B and C: New Diterpenes from Dictyota dichotoma Hudson
Source: Mar Drugs. 2013 Aug 22;11(9):3109–23. doi: 10.3390/md11093109 (PMC3801117; doi:10.3390/md11093109)
Supplement: Supplementary File 1 — Supplementary Information (PDF, 4646 KB) [file marinedrugs-11-03109-s001.pdf]

Supplementary Information

Figure S1. ^{1}H NMR spectrum ($CDCl_3$, 300 MHz) of *cis*-pachydictyol B (**1a**) 2
Figure S2. ^{13}C NMR Spectrum ($CDCl_3$, 125 MHz) of *cis*-Pachydictyol B (**1a**) 3
Figure S3. ^{1}H–^{1}H-COSY Spectrum ($CDCl_3$, 300 MHz) of *cis*-Pachydictyol B (**1a**) 4
Figure S4. HSQC spectrum ($CDCl_3$, 300 MHz) of *cis*-Pachydictyol B (**1a**) 5
Figure S5. HMBC Spectrum ($CDCl_3$, 300 MHz) of *cis*-Pachydictyol B (**1a**) 6
Figure S6. NOESY Spectrum ($CDCl_3$, 600 MHz) of *cis*-Pachydictyol B (**1a**) 7
Figure S7. ^{1}H NMR Spectrum ($CDCl_3$, 600 MHz) of *trans*-Pachydictyol B (**1b**) 8
Figure S8. H,H COSY Spectrum ($CDCl_3$, 600 MHz) of *trans*-Pachydictyol B (**1b**) 9
Figure S9. HSQC Spectrum ($CDCl_3$, 600/150 MHz) of *trans*-Pachydictyol B (**1b**) 10
Figure S10. HMBC Spectrum ($CDCl_3$, 600/150 MHz) of *trans*-Pachydictyol B (**1b**) 11
Figure S11. HMBC correlations in *trans*-pachydictyol B (**1b**) 12
Figure S12. ^{1}H NMR Spectrum ($CDCl_3$, 300 MHz) of Pachydictyol C (**2**) 13
Figure S13. ^{13}C NMR spectrum ($CDCl_3$, 125 MHz) of Pachydictyol C (**2**) 14
Figure S14. ^{1}H–^{1}H-COSY spectrum ($CDCl_3$, 300 MHz) of Pachydictyol C (**2**) 15
Figure S15. HSQC spectrum ($CDCl_3$, 300 MHz) of Pachydictyol C (**2**) 16
Figure S16. HMBC spectrum ($CDCl_3$, 300 MHz) of Pachydictyol C (**2**) 17
Figure S17. ^{1}H NMR spectrum ($CDCl_3$, 300 MHz) of Pachydictyol A (**3**) 18
Figure S18. ^{13}C NMR Spectrum ($CDCl_3$, 125 MHz) of Pachydictyol A (**3**) 19
Figure S19. ^{1}H–^{1}H-COSY Spectrum ($CDCl_3$, 300 MHz) of Pachydictyol A (**3**) 20
Figure S20. HSQC Spectrum ($CDCl_3$, 300 MHz) of Pachydictyol A (**3**) 21
Figure S21. HMBC Spectrum ($CDCl_3$, 300 MHz) of Pachydictyol A (**3**) 22
Figure S22. ^{1}H NMR Spectrum ($CDCl_3$, 300 MHz) of Dictyol E (**4**) 23
Figure S23. ^{13}C NMR Spectrum ($CDCl_3$, 125 MHz) of Dictyol E (**4**) 24
Figure S24. ^{1}H–^{1}H-COSY Spectrum ($CDCl_3$, 300 MHz) of Dictyol E (**4**) 25
Figure S25. HSQC Spectrum ($CDCl_3$, 300 MHz) of Dictyol E (**4**) 26
Figure S26. HMBC Spectrum ($CDCl_3$, 300 MHz) of Dictyol E (**4**) 27
Figure S27. NOESY Spectrum ($CDCl_3$, 600 MHz) of Dictyol E (**4**) 28
Figure S28. ^{1}H NMR Spectrum ($CDCl_3$, 300 MHz) of *cis*-Africanan-1α-ol (**5a**) 29
Figure S29. ^{13}C NMR Spectrum ($CDCl_3$, 125 MHz) of *cis*-Africanan-1α-ol (**5a**) 30
Figure S30. ^{1}H–^{1}H-COSY Spectrum ($CDCl_3$, 300 MHz) of *cis*-Africanan-1α-ol (**5a**) 31
Figure S31. HSQC Spectrum ($CDCl_3$, 300 MHz) of *cis*-Africanan-1α-ol (**5a**) 32
Figure S32. HMBC Spectrum ($CDCl_3$, 300 MHz) of *cis*-Africanan-1α-ol (**5a**) 33
Figure S33. ^{1}H NMR Spectrum ($CDCl_3$, 300 MHz) of Fucosterol (**6**) 34
Figure S34. ^{13}C NMR Spectrum ($CDCl_3$, 125 MHz) of Fucosterol (**6**) 35
Table S1. GC-MS analysis of the nonpolar fraction I 36
Table S2. GC-MS analysis of the unsaponifiable part of the petroleum ether extract of *D. dichotoma* 36

Figure S1. ^{1}H NMR spectrum ($CDCl_3$, 300 MHz) of *cis*-pachydictyol B (**1a**).

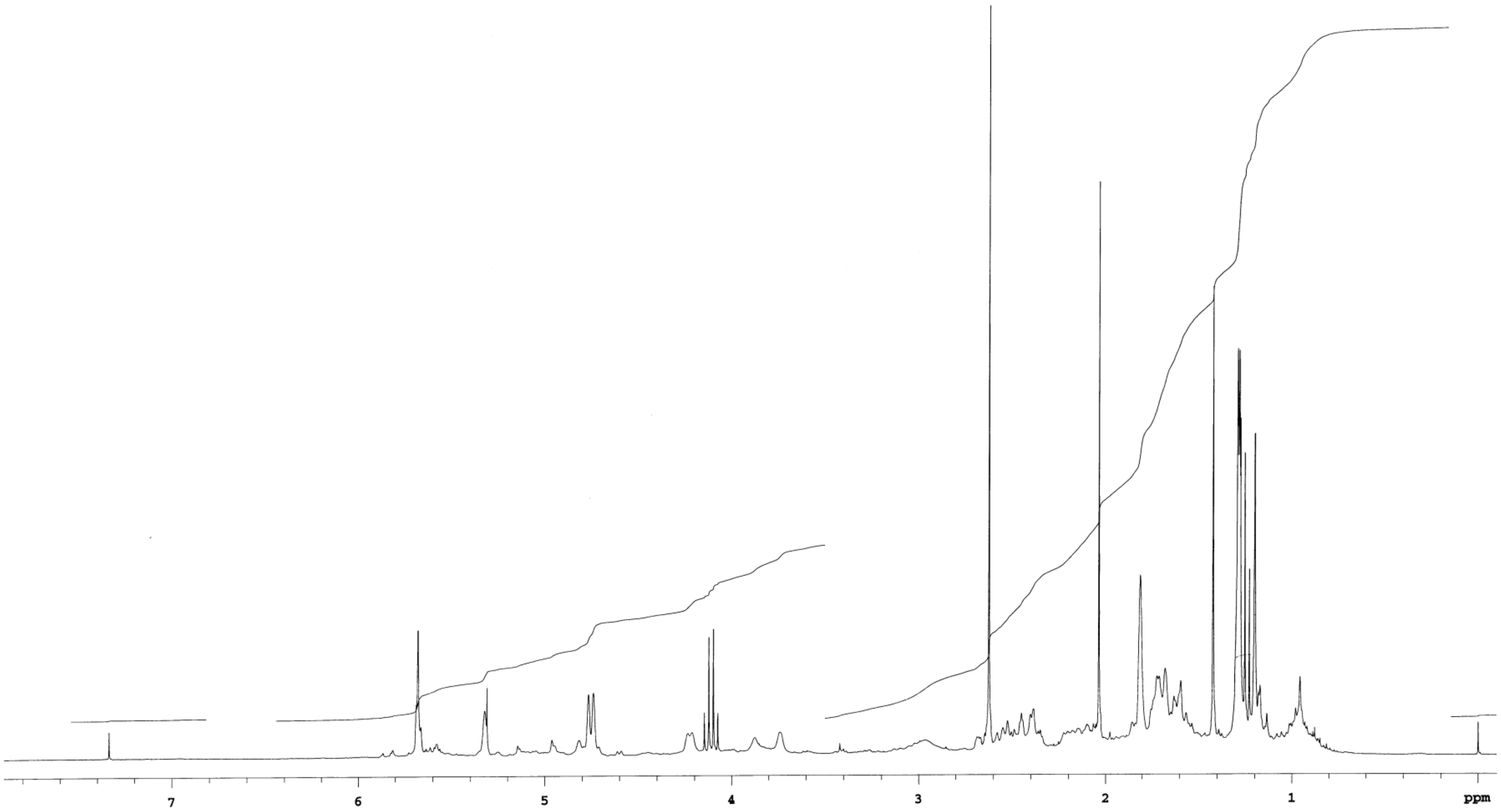

Figure S2. ^{13}C NMR spectrum ($CDCl_3$, 125 MHz) of *cis*-pachydictyol B (**1a**).

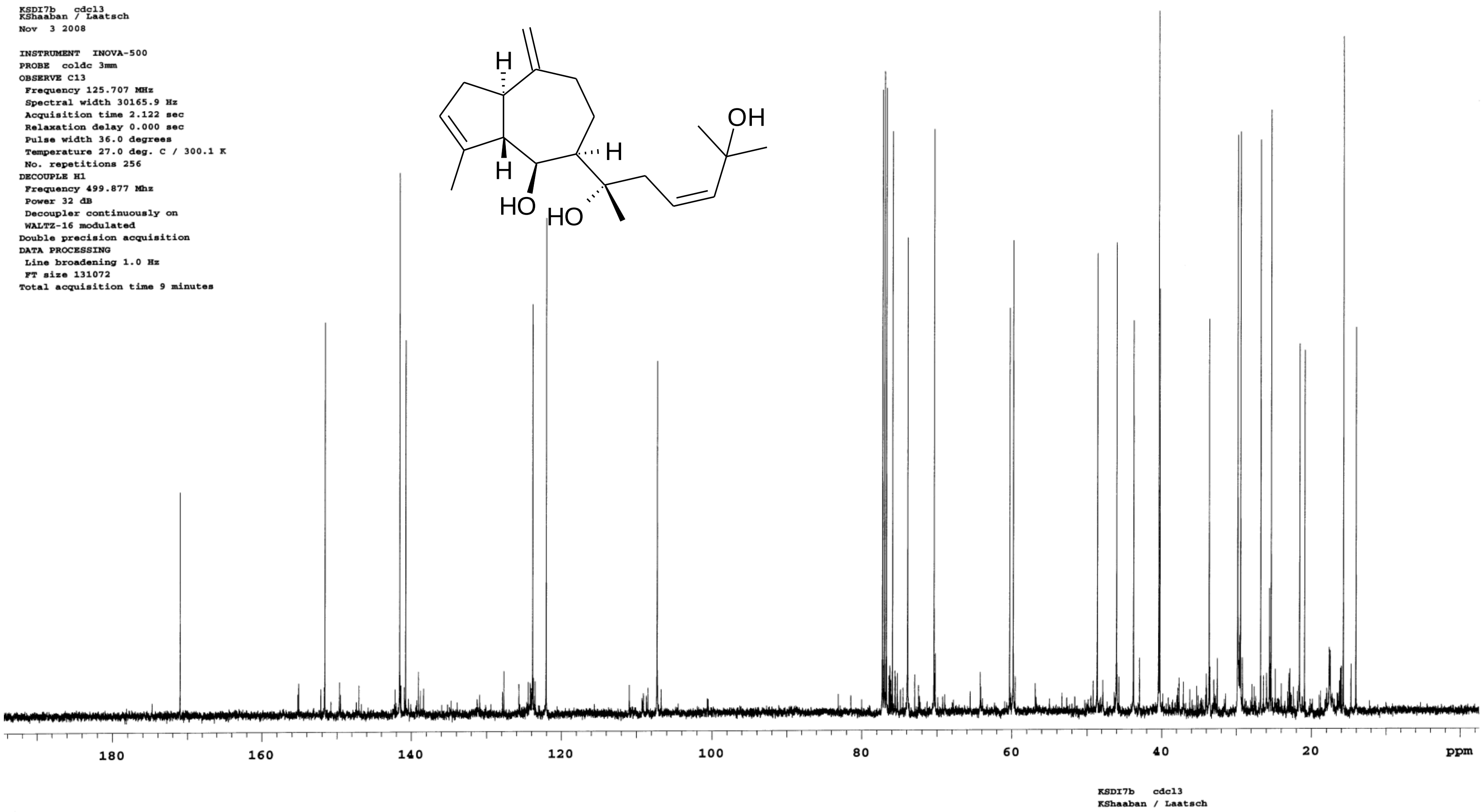

The carbon signals at δ 171.0, 60.3, 20.9 und 14.1 are belonging to ethyl acetate (ethyl acetate present in the deuterated $CDCl_3$), and the signal δ 26.8due to fatty acid impurities.

Figure S3. ^{1}H–^{1}H-COSY spectrum ($CDCl_3$, 300 MHz) of *cis*-pachydictyol B (**1a**).

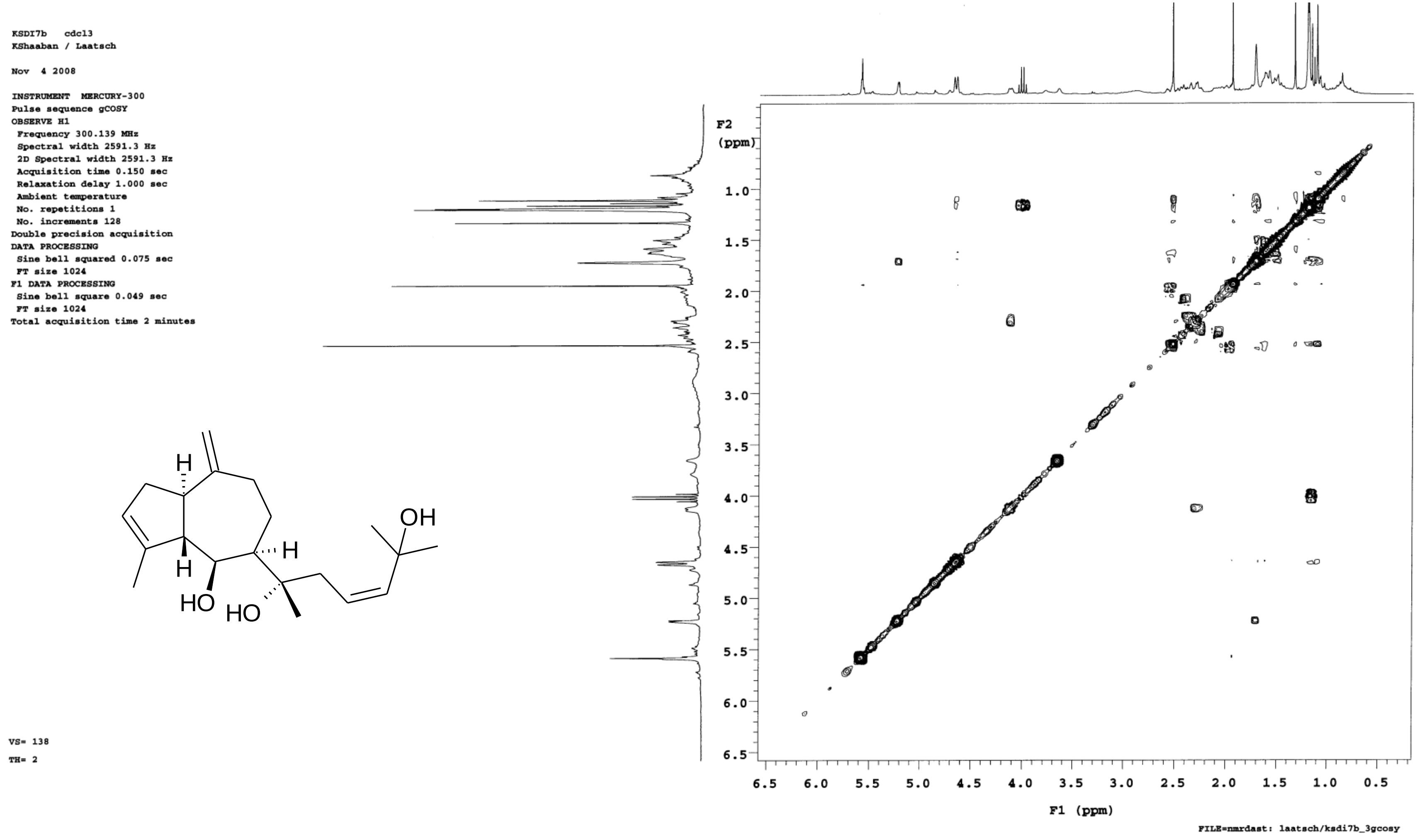

Figure S4. HSQC spectrum ($CDCl_3$, 300 MHz) of *cis*-pachydictyol B (**1a**).

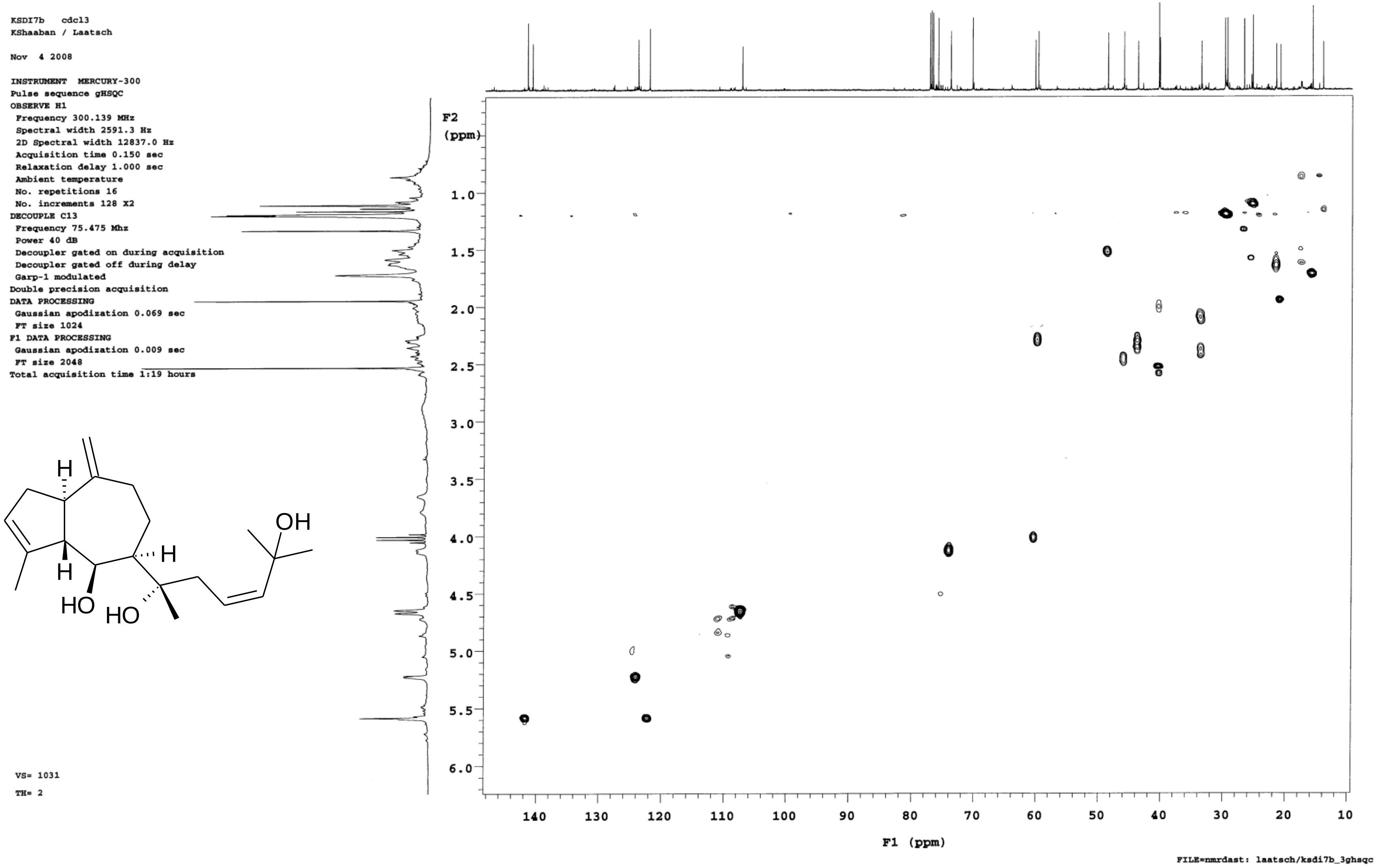

Figure S5. HMBC spectrum ($CDCl_3$, 300 MHz) of *cis*-pachydictyol B (**1a**).

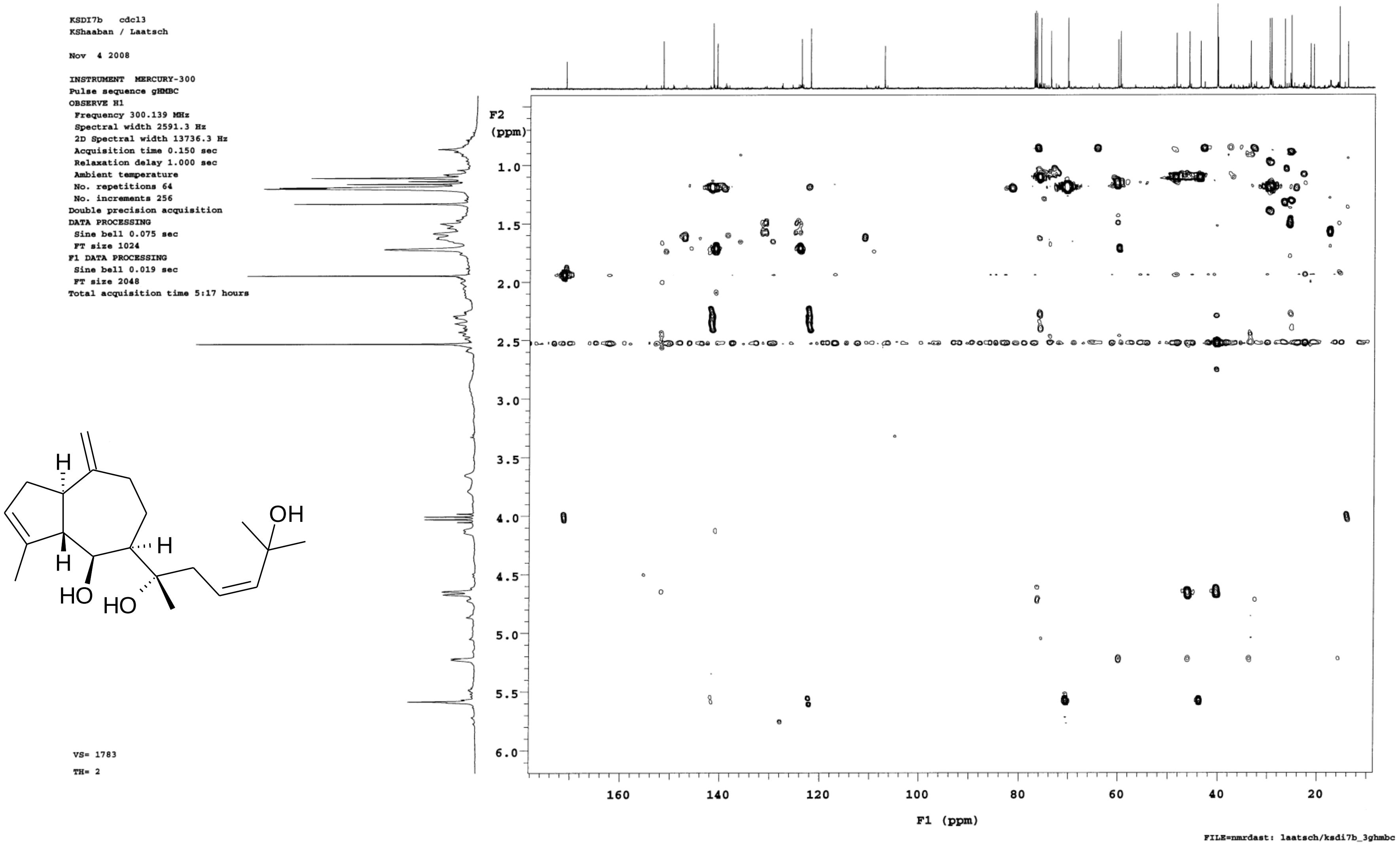

Figure S6. NOESY spectrum ($CDCl_3$, 600 MHz) of *cis*-pachydictyol B (**1a**).

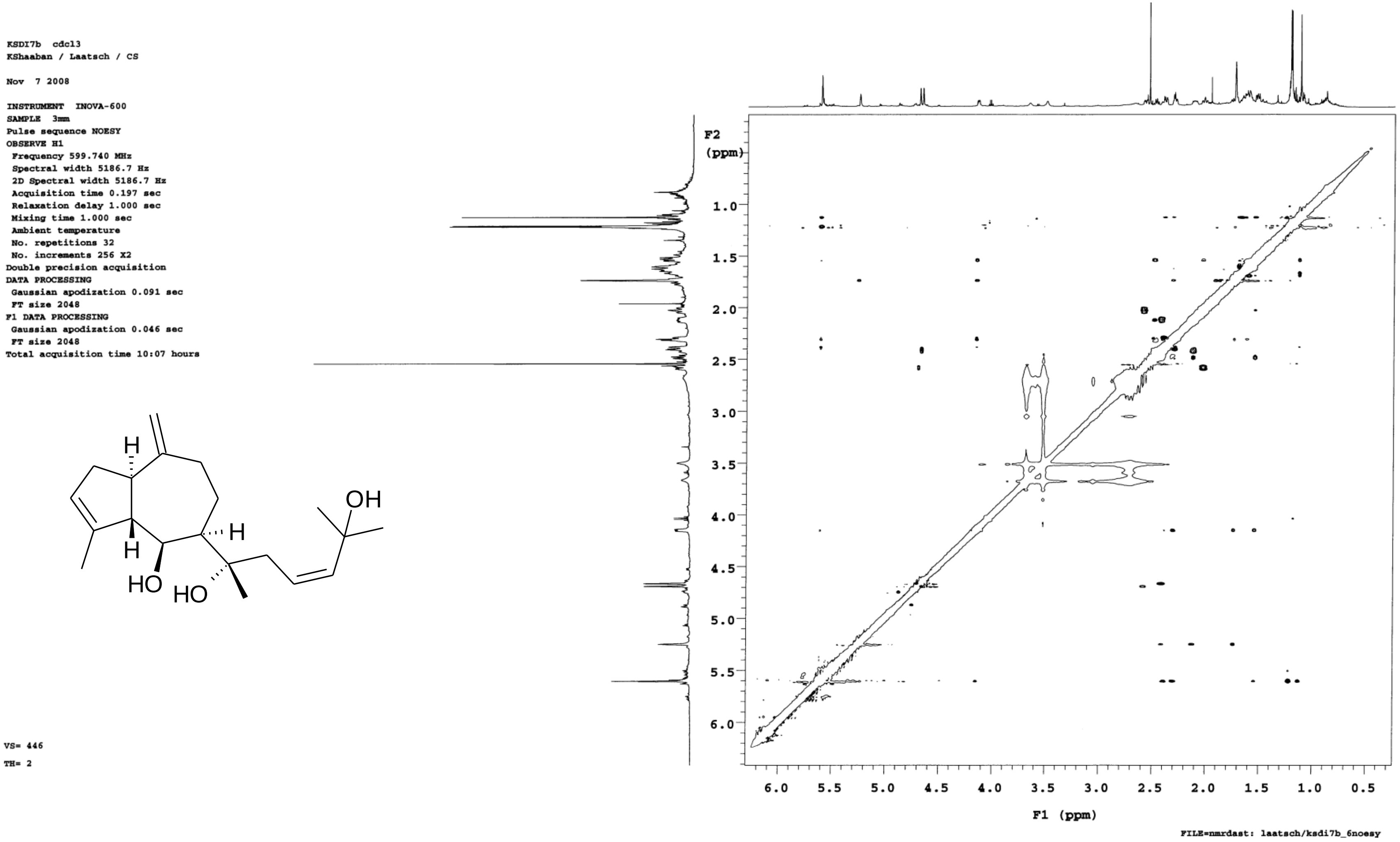

Figure S7. ^{1}H NMR spectrum ($CDCl_3$, 600 MHz) of *trans*-pachydictyol B (**1b**).

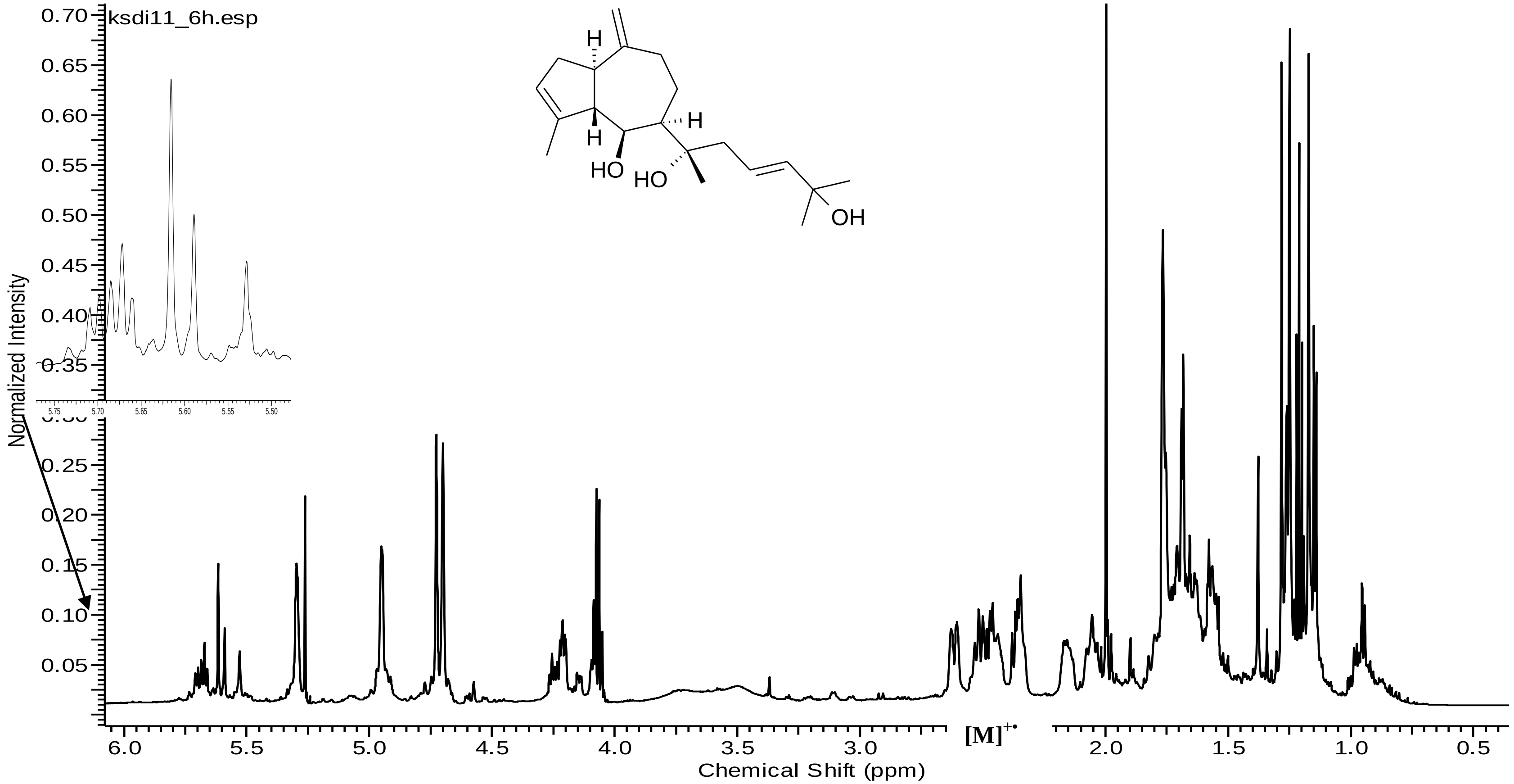

Figure S8. H,H COSY spectrum ($CDCl_3$, 600 MHz) of *trans*-pachydictyol B (**1b**).

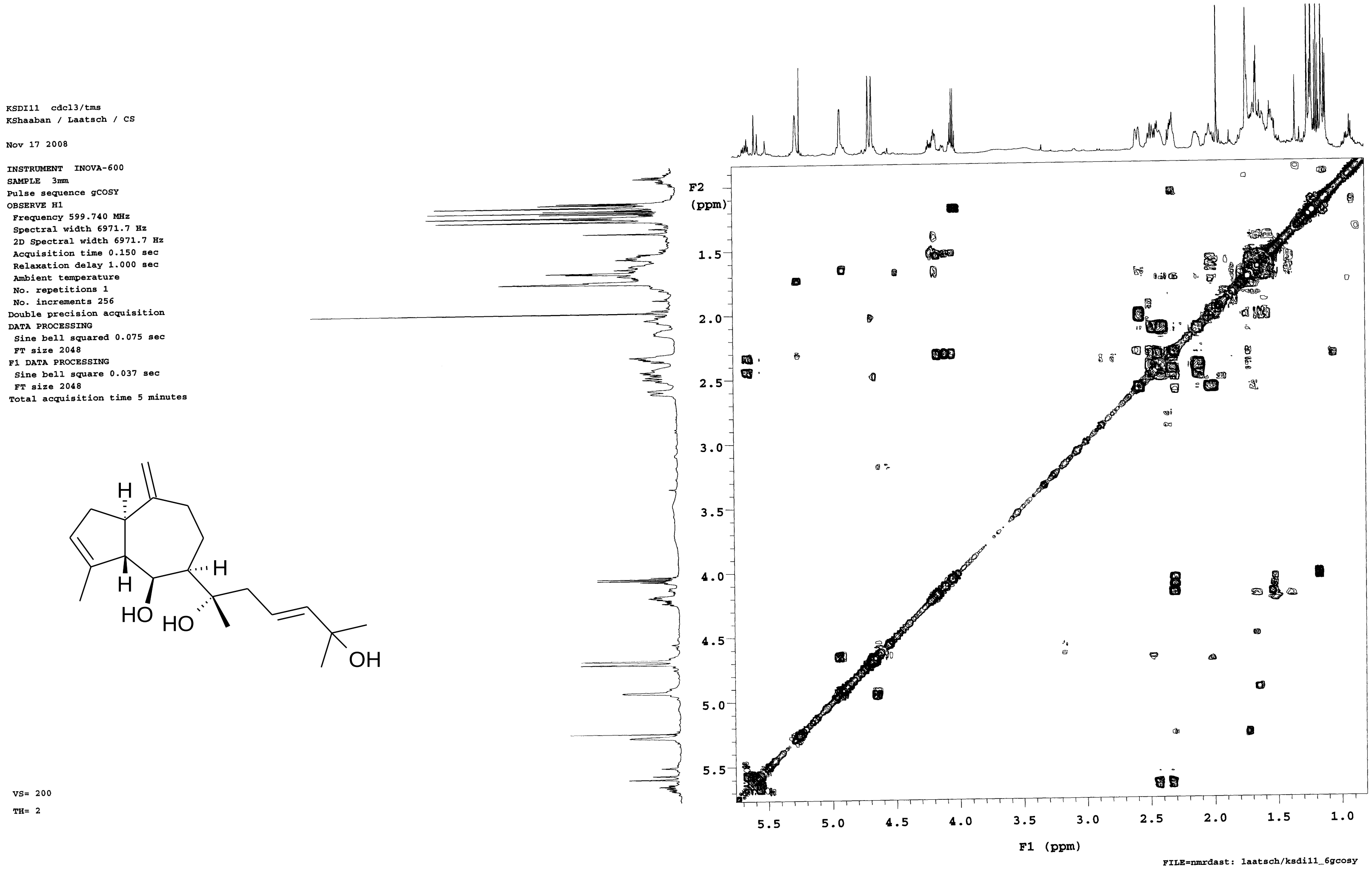

Figure S9. HSQC spectrum ($CDCl_3$, 600/150 MHz) of *trans*-pachydictyol B (**1b**).

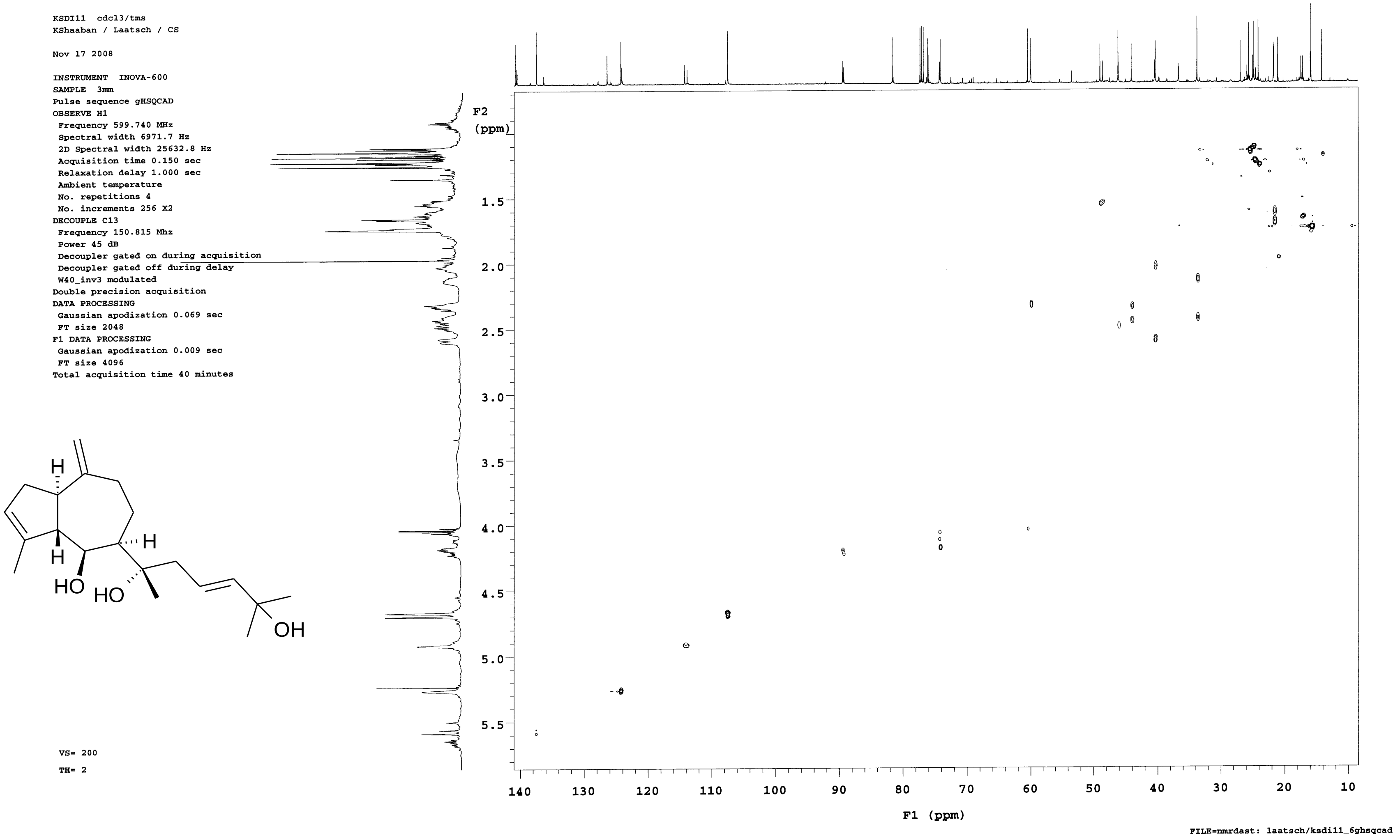

Figure S10. HMBC spectrum ($CDCl_3$, 600/150 MHz) of *trans*-pachydictyol B (**1b**).

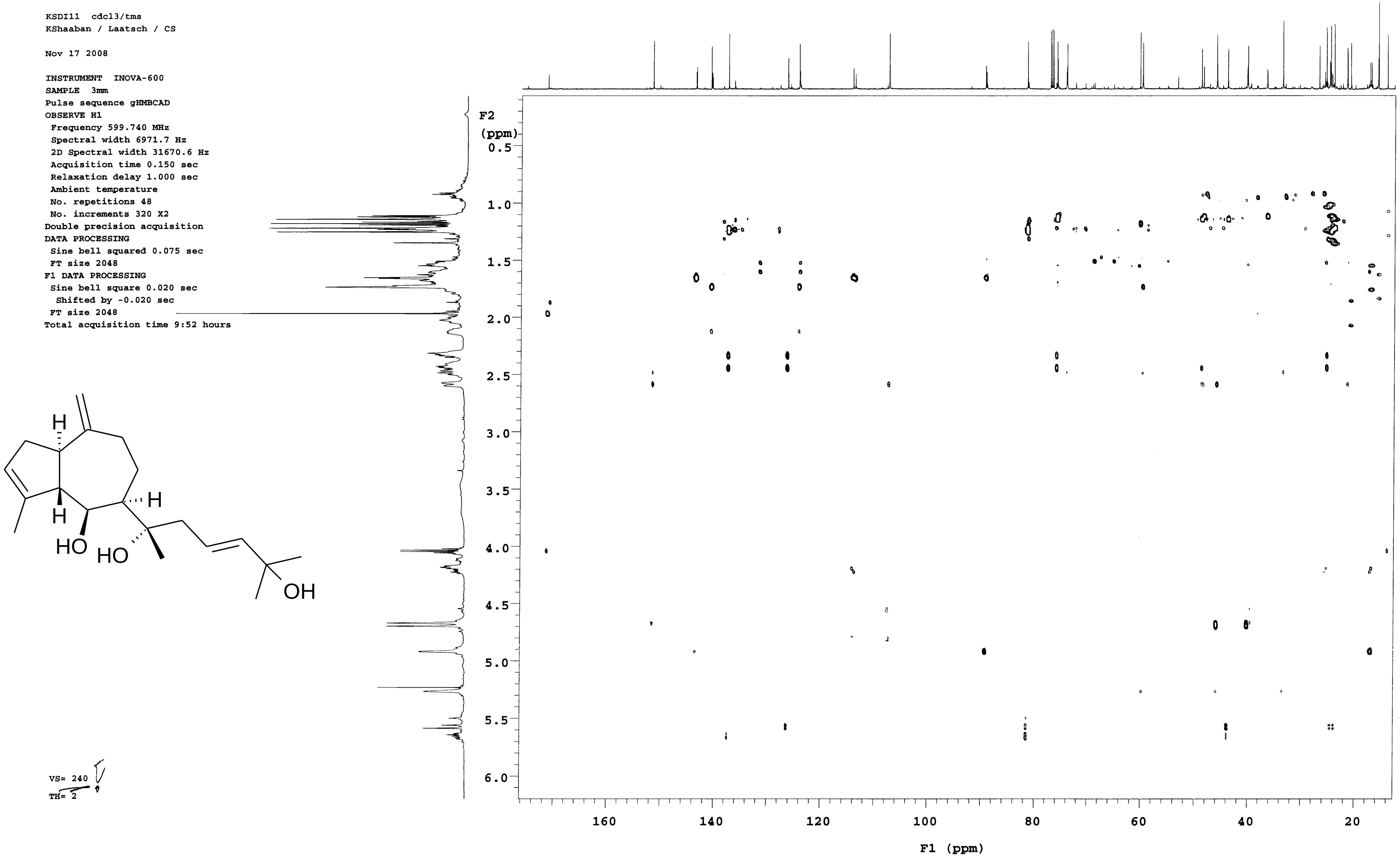

Figure S11. HMBC correlations in *trans*-pachydictyol B (**1b**).

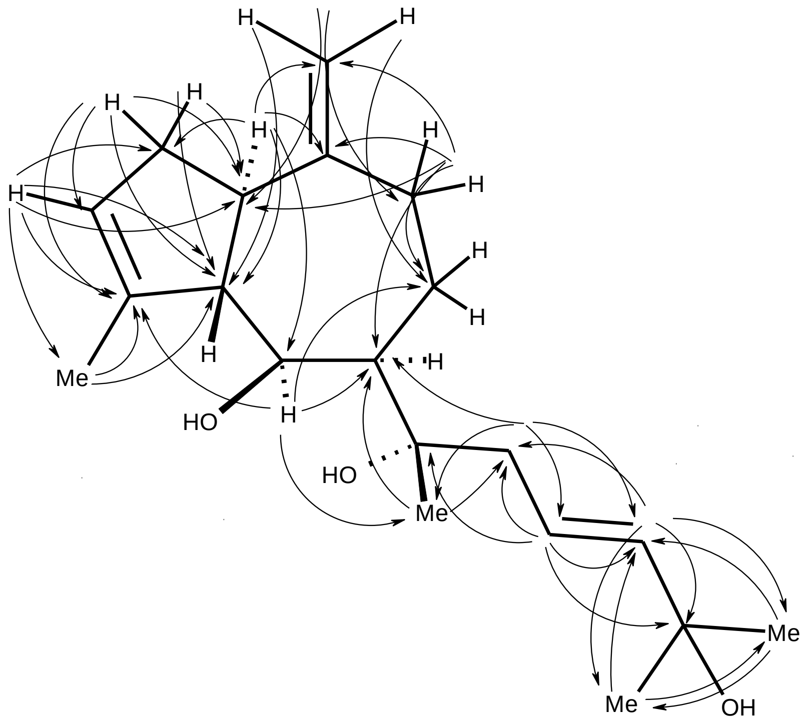

Figure S12. ^{1}H NMR spectrum ($CDCl_3$, 300 MHz) of pachydictyol C (**2**).

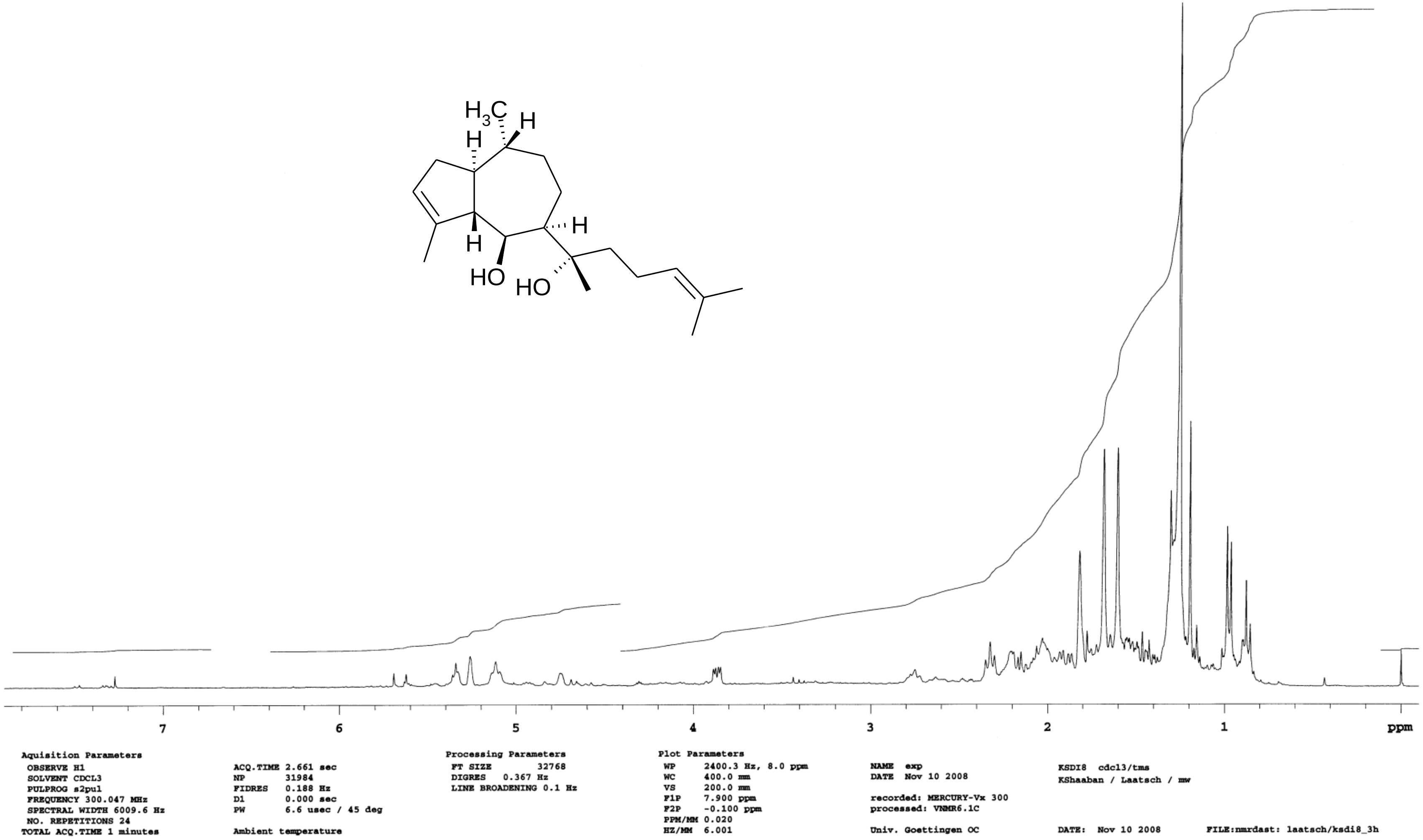

Figure S13. ^{13}C NMR spectrum ($CDCl_3$, 125 MHz) of pachydictyol C (**2**).

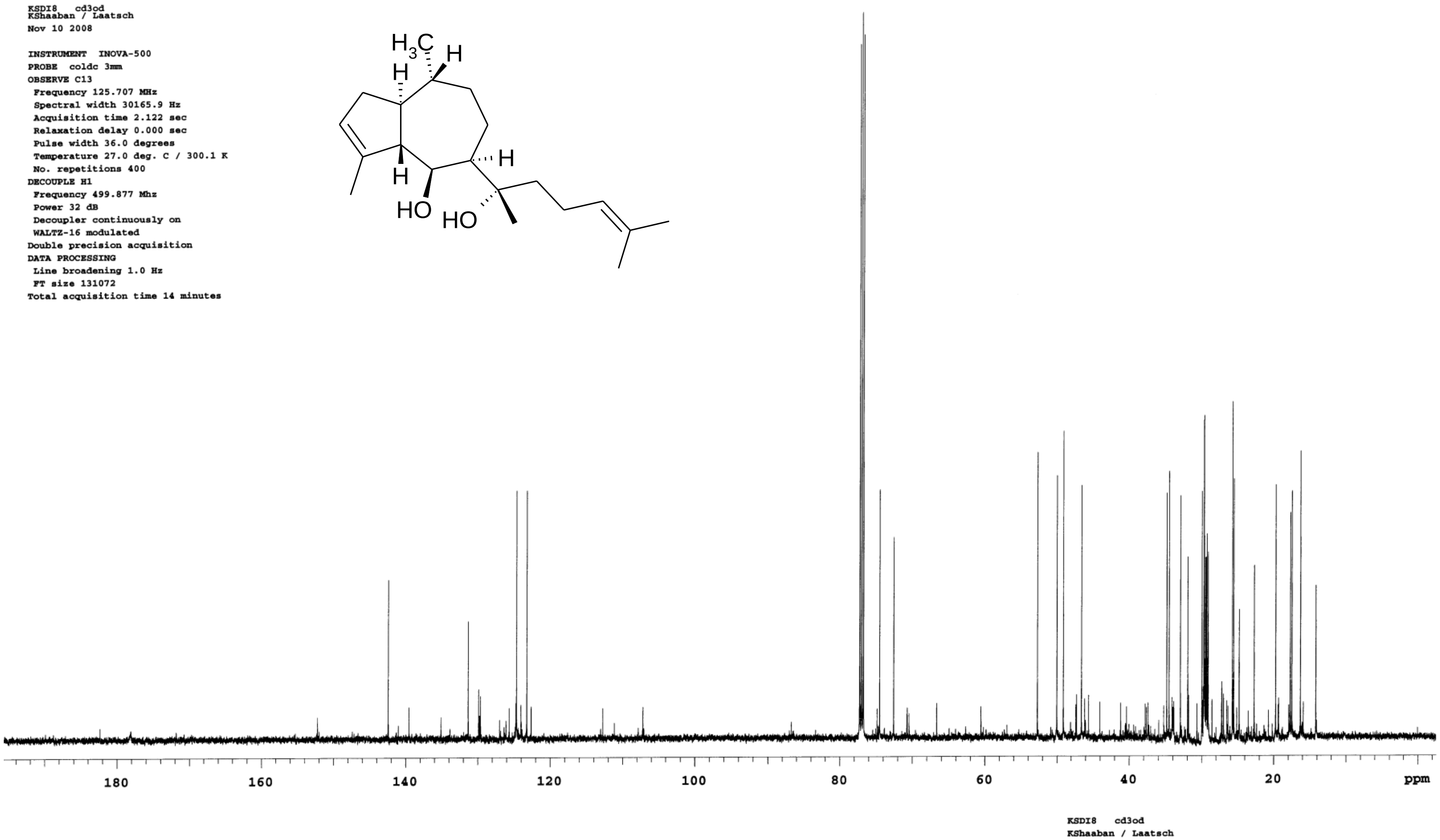

Figure S14. ^{1}H–^{1}H-COSY spectrum ($CDCl_3$, 300 MHz) of pachydictyol C (**2**).

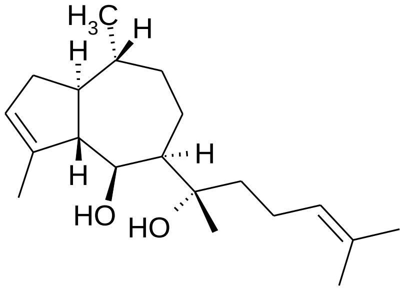

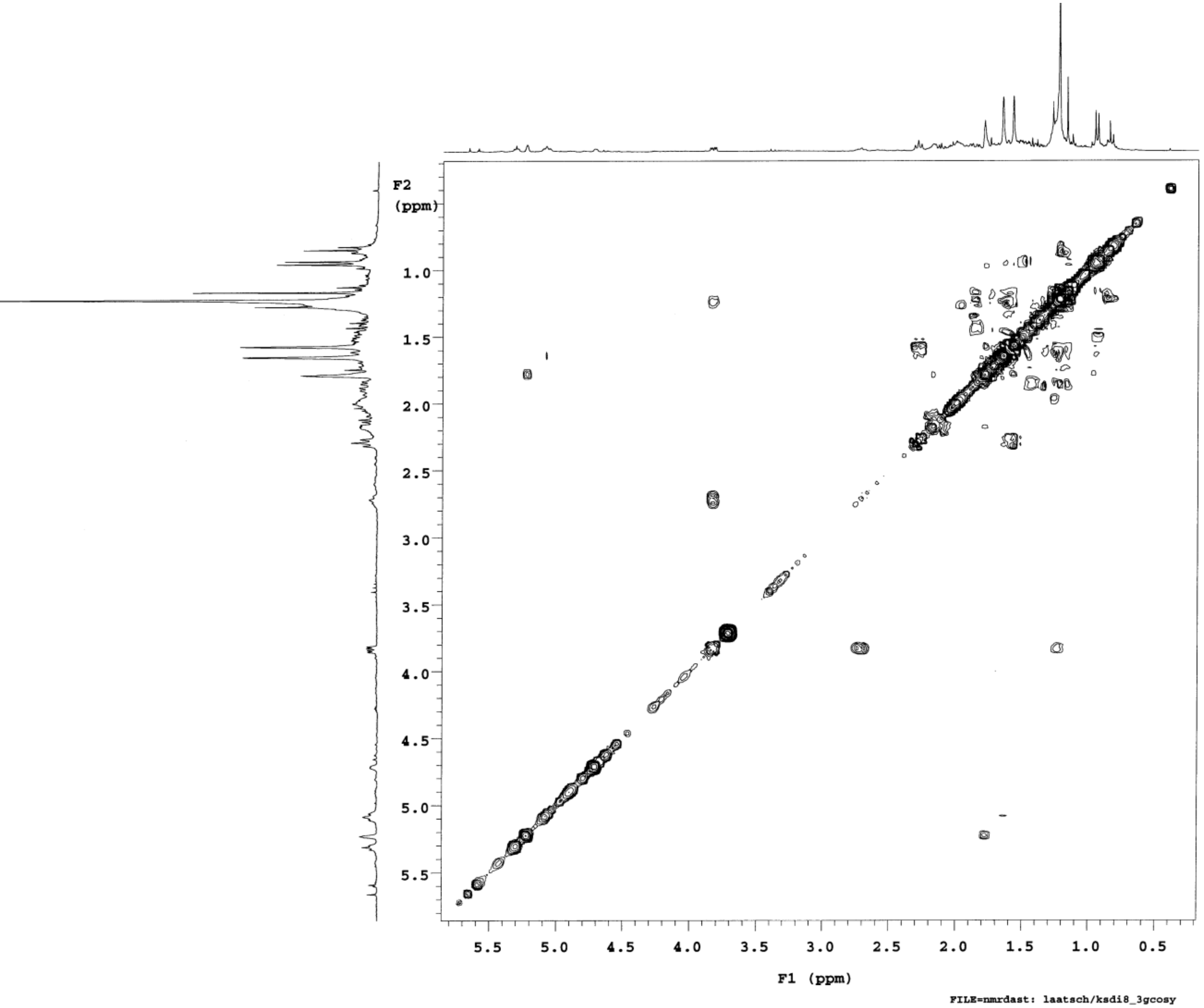

Figure S15. HSQC spectrum ($CDCl_3$, 300 MHz) of pachydictyol C (**2**).

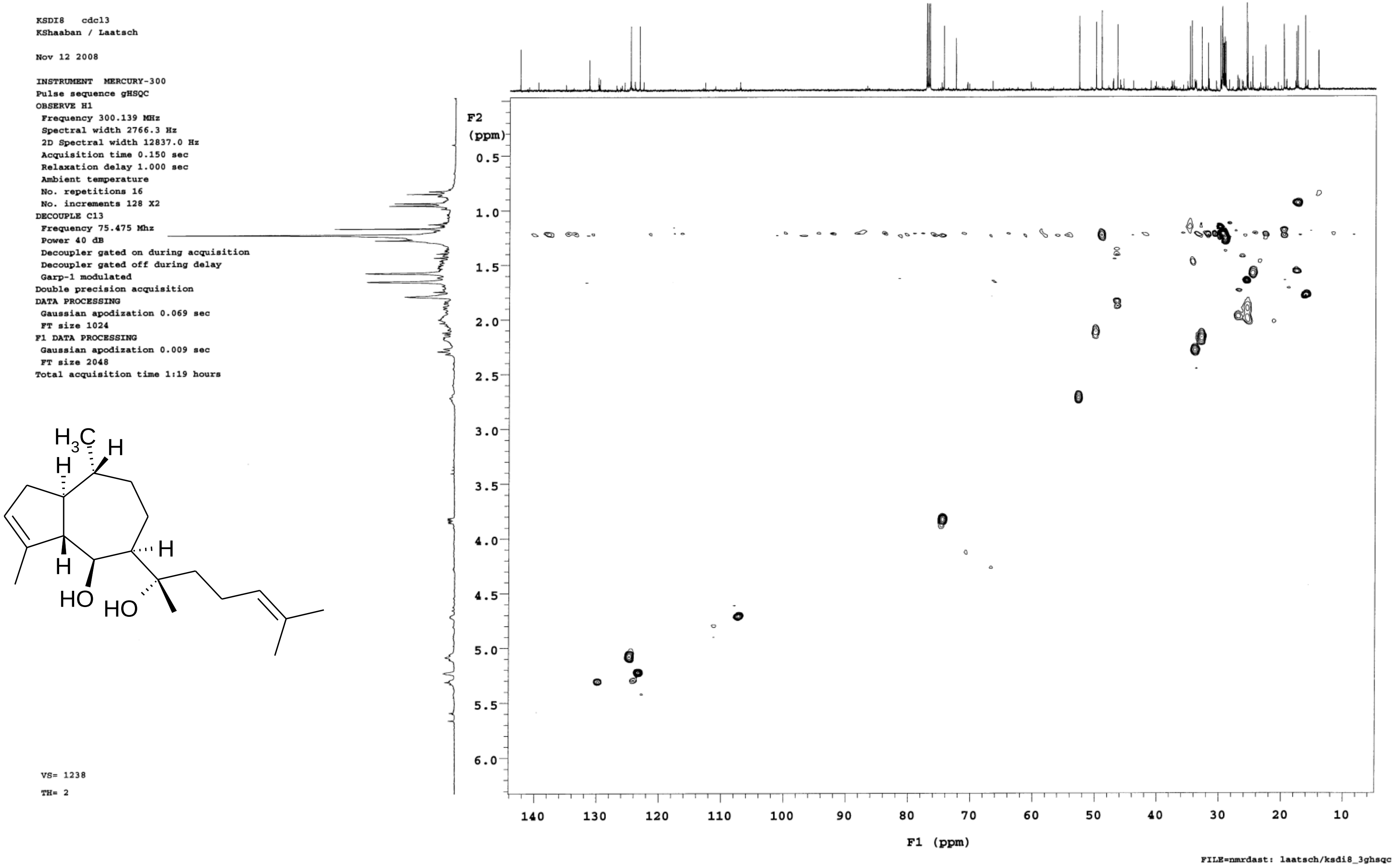

Figure S16. HMBC spectrum ($CDCl_3$, 300 MHz) of pachydictyol C (**2**).

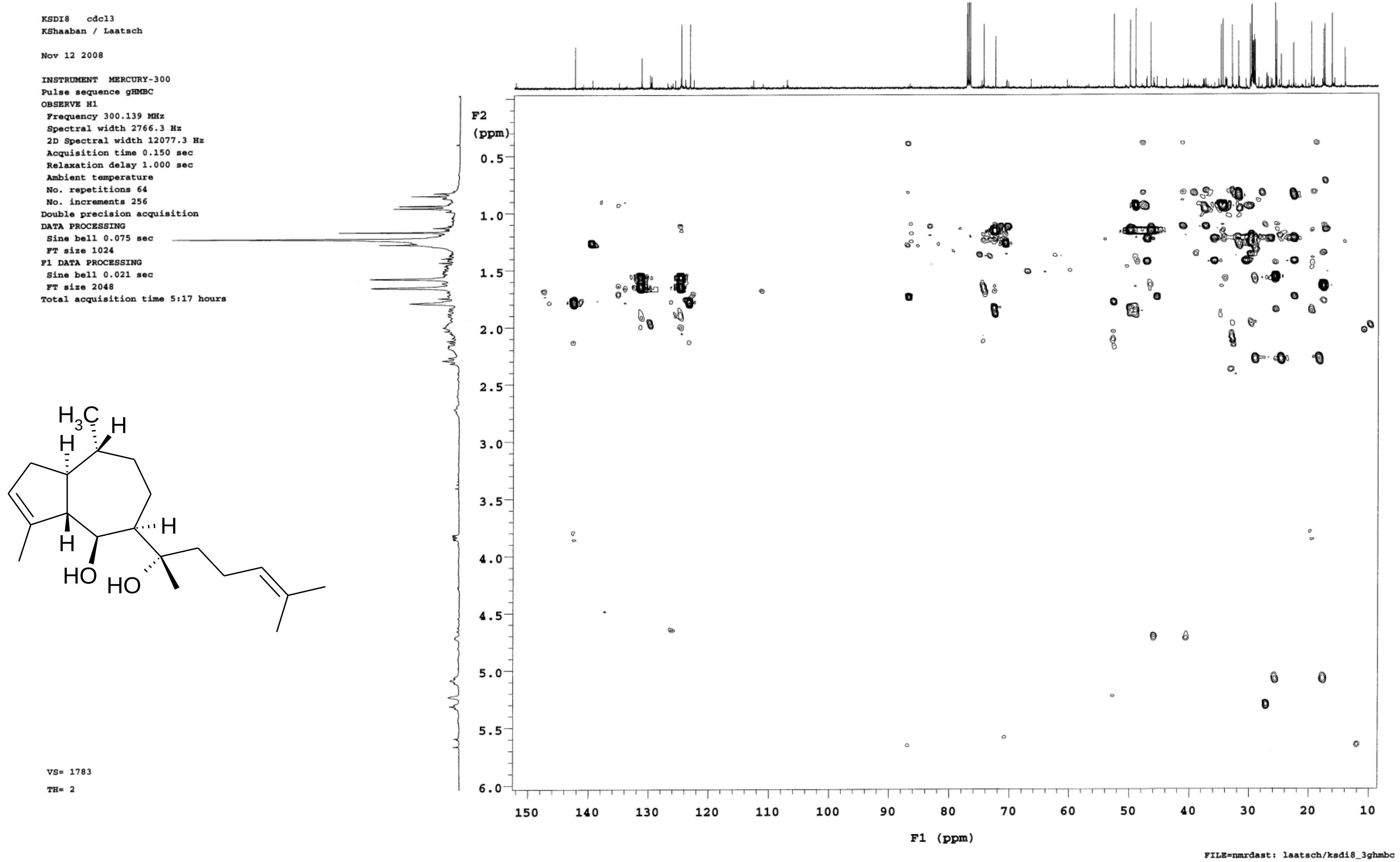

Figure S17. ^{1}H NMR spectrum ($CDCl_3$, 300 MHz) of pachydictyol A (**3**).

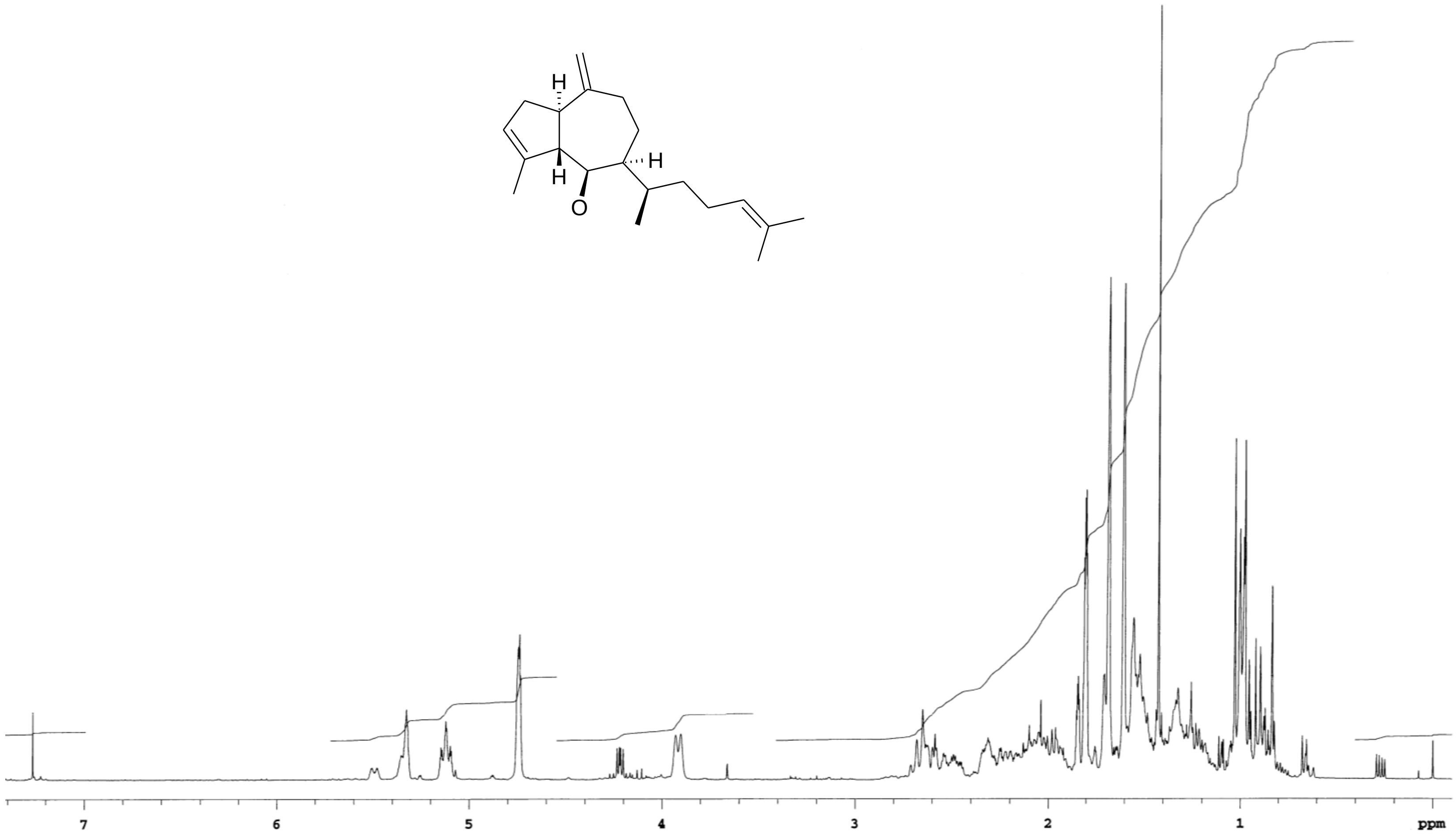

Figure S18. ^{13}C NMR spectrum ($CDCl_3$, 125 MHz) of pachydictyol A (**3**).

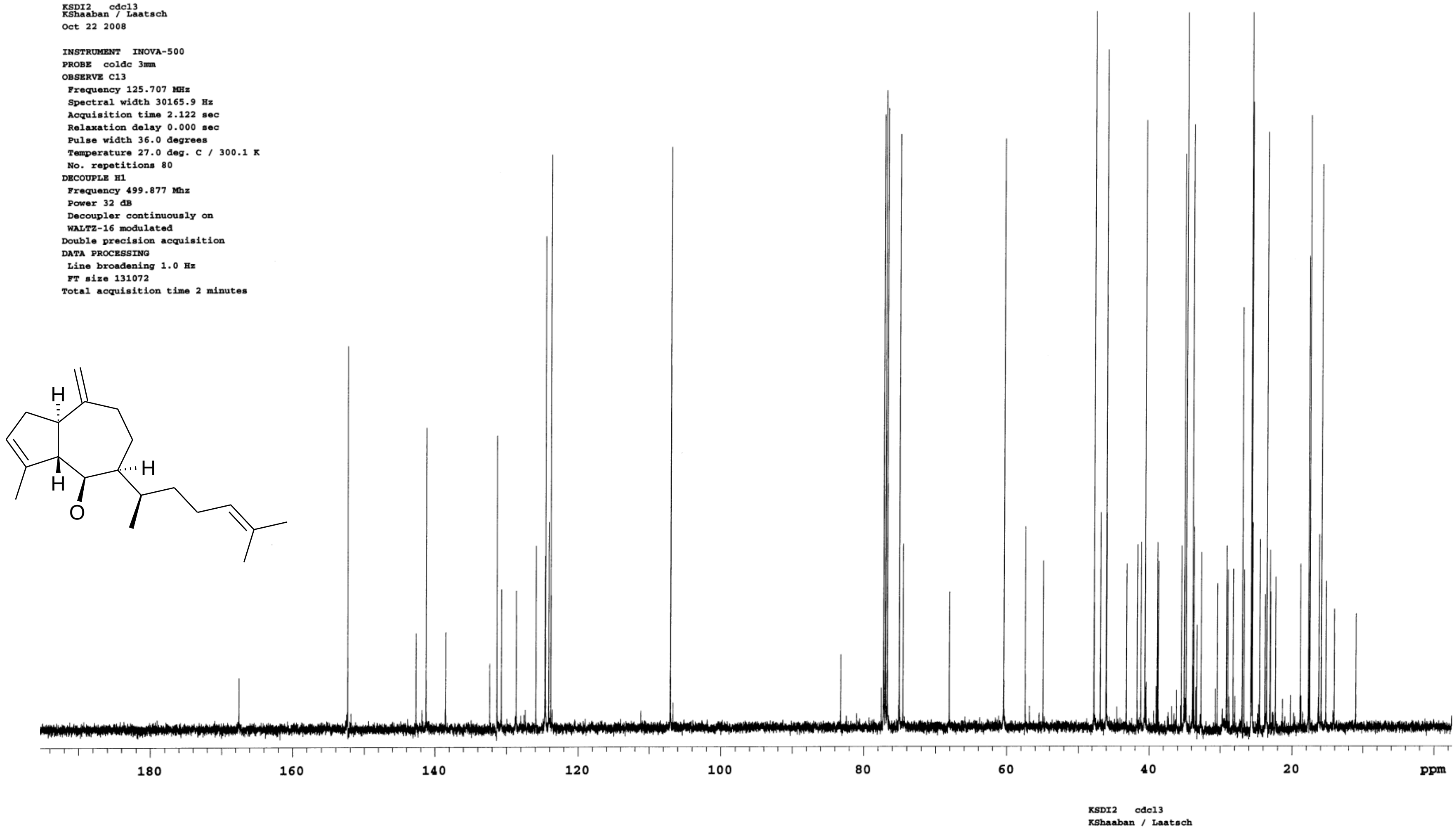

Figure S19. ^{1}H–^{1}H-COSY spectrum ($CDCl_3$, 300 MHz) of pachydictyol A (**3**).

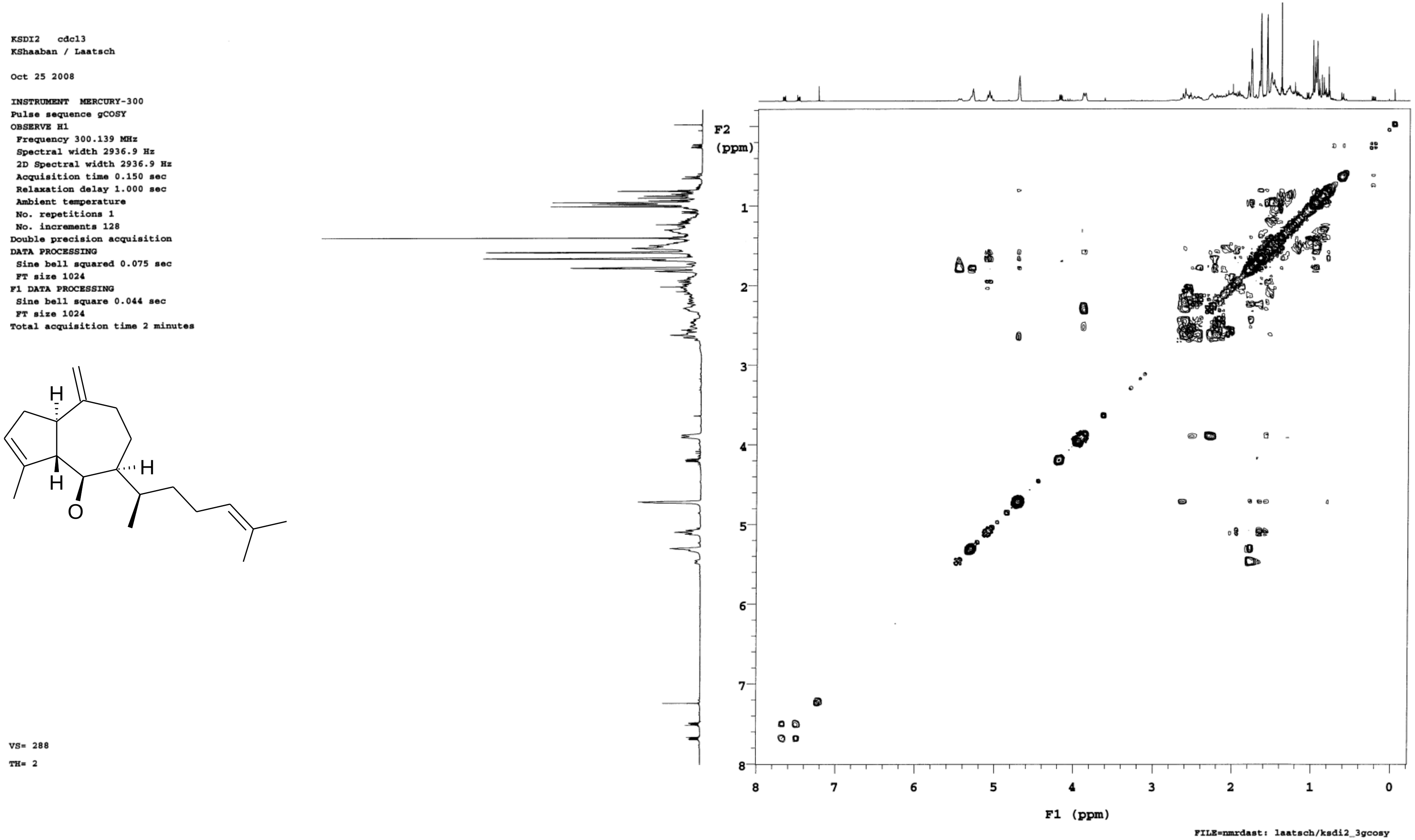

Figure S20. HSQC spectrum ($CDCl_3$, 300 MHz) of pachydictyol A (**3**).

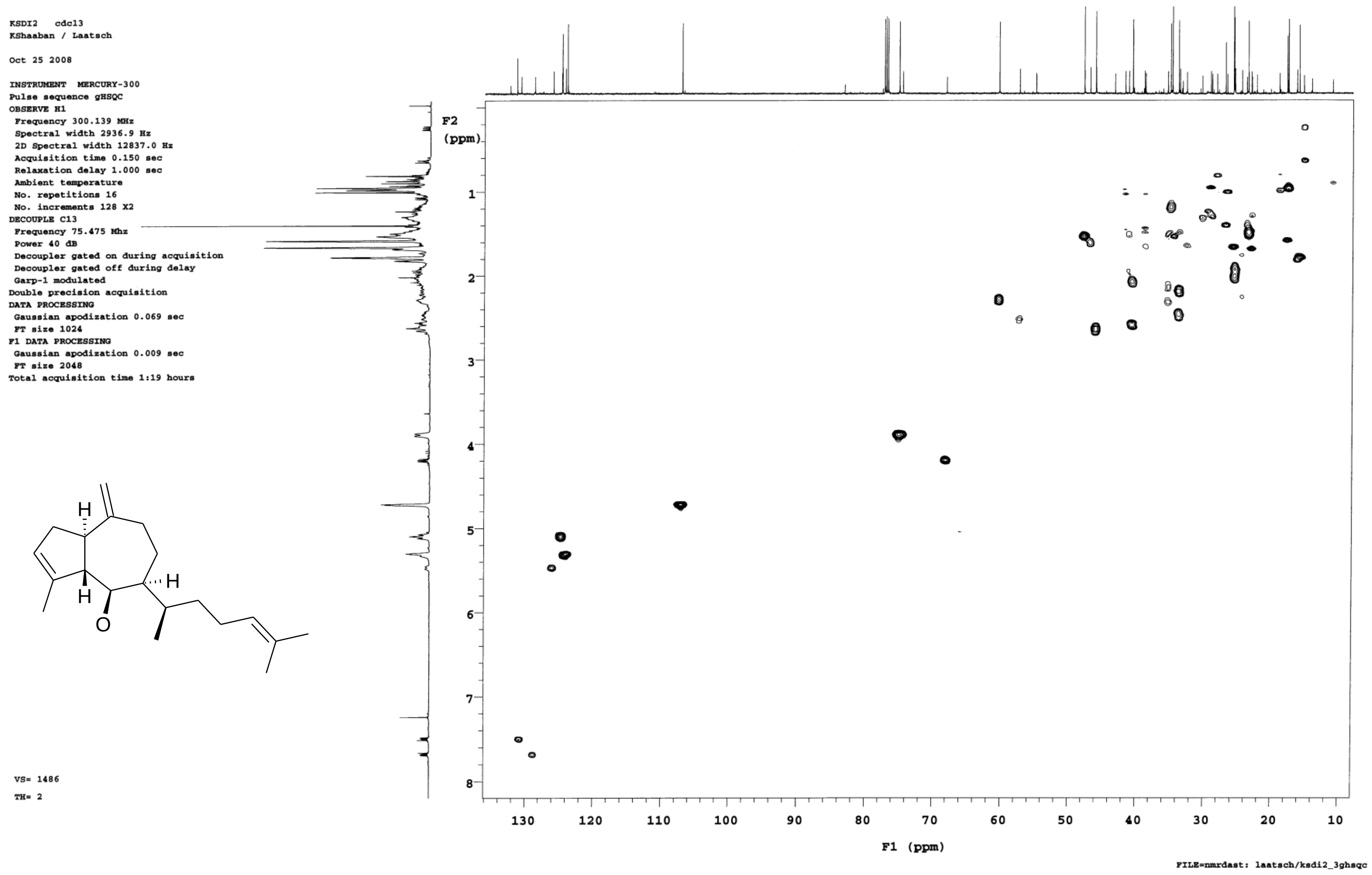

Figure S21. HMBC spectrum ($CDCl_3$, 300 MHz) of pachydictyol A (**3**).

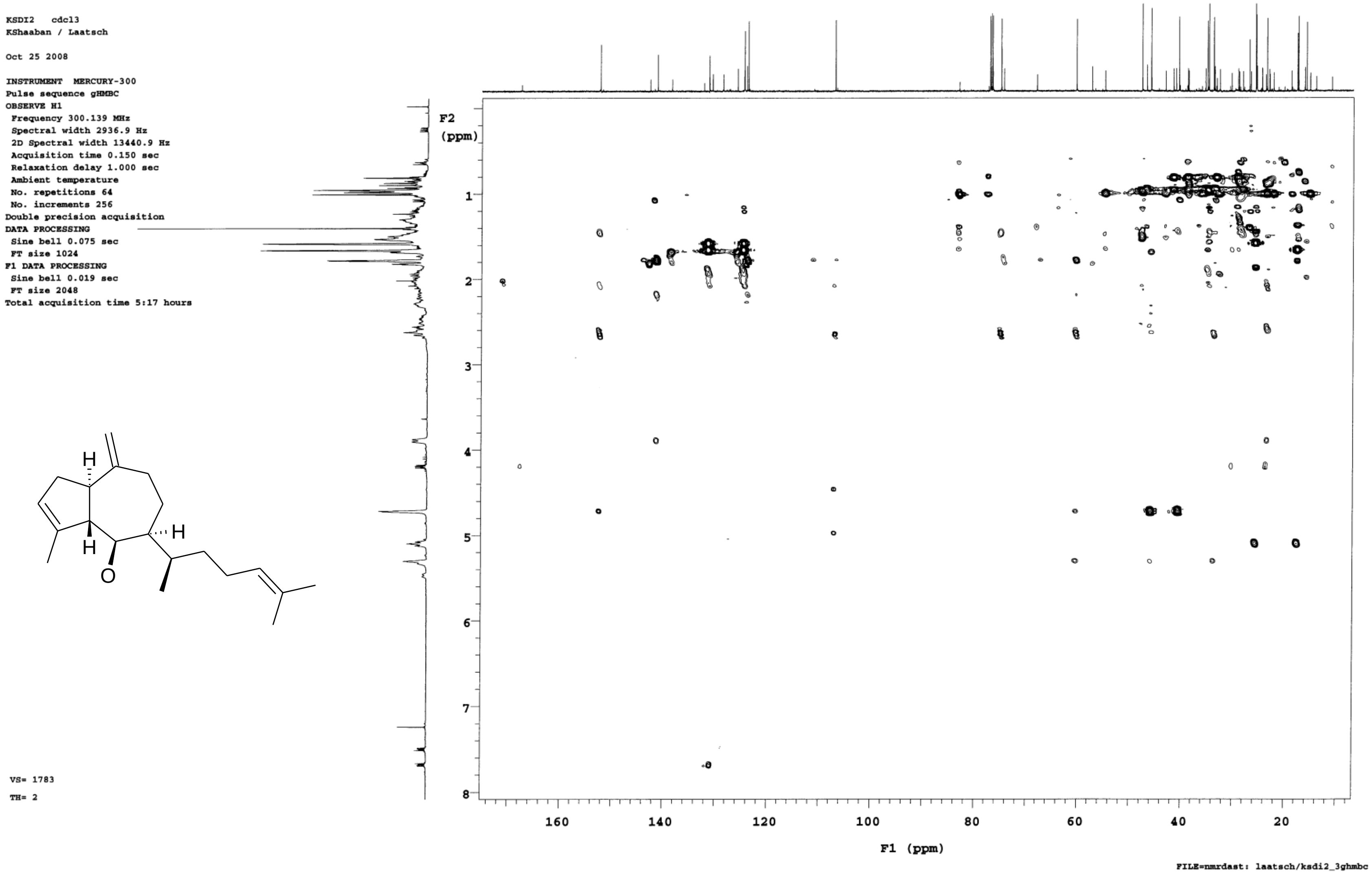

Figure S22. ^{1}H NMR spectrum ($CDCl_3$, 300 MHz) of dictyol E (**4**).

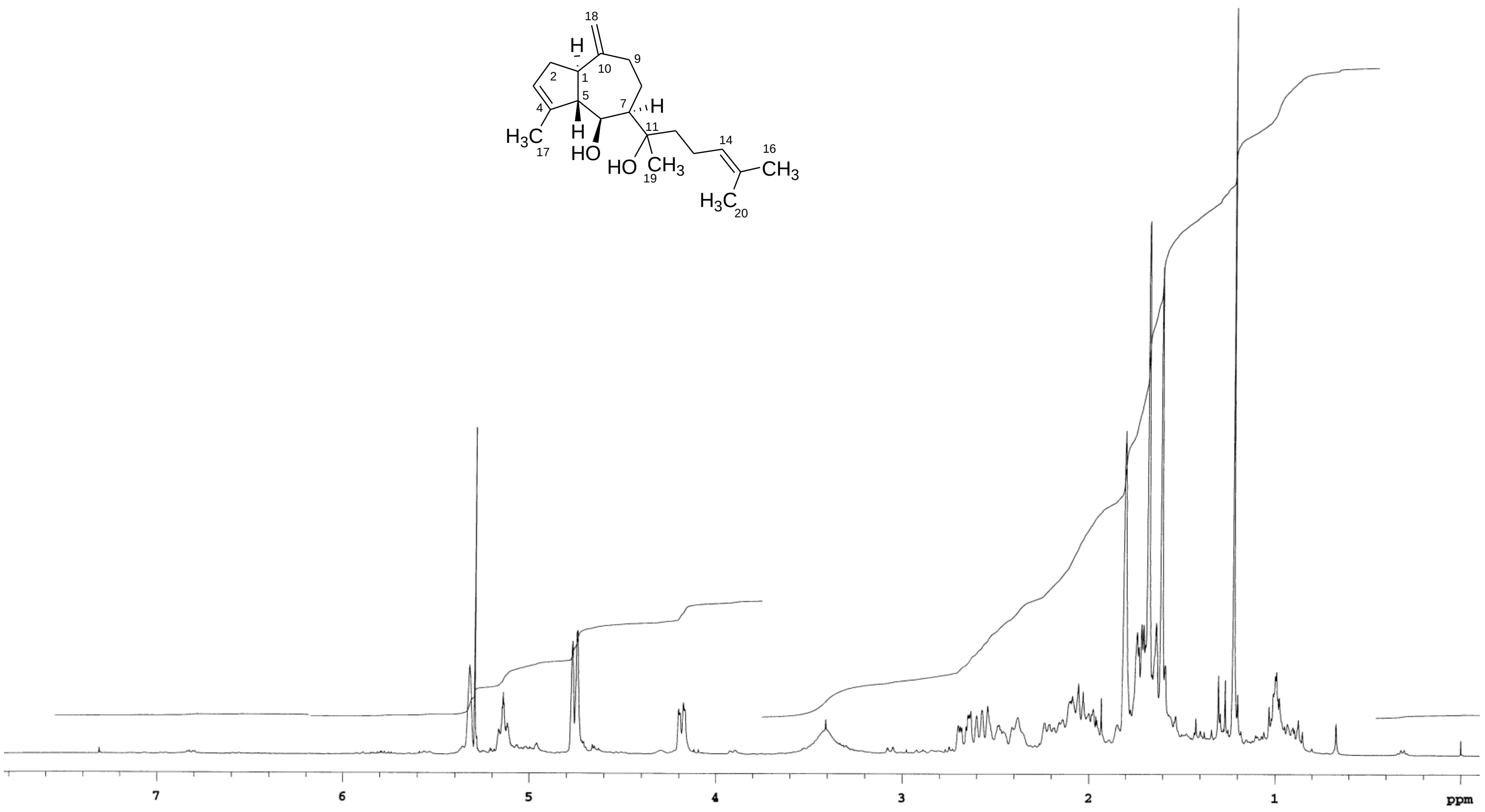

Figure S23. ^{13}C NMR spectrum ($CDCl_3$, 125 MHz) of dictyol E (**4**).

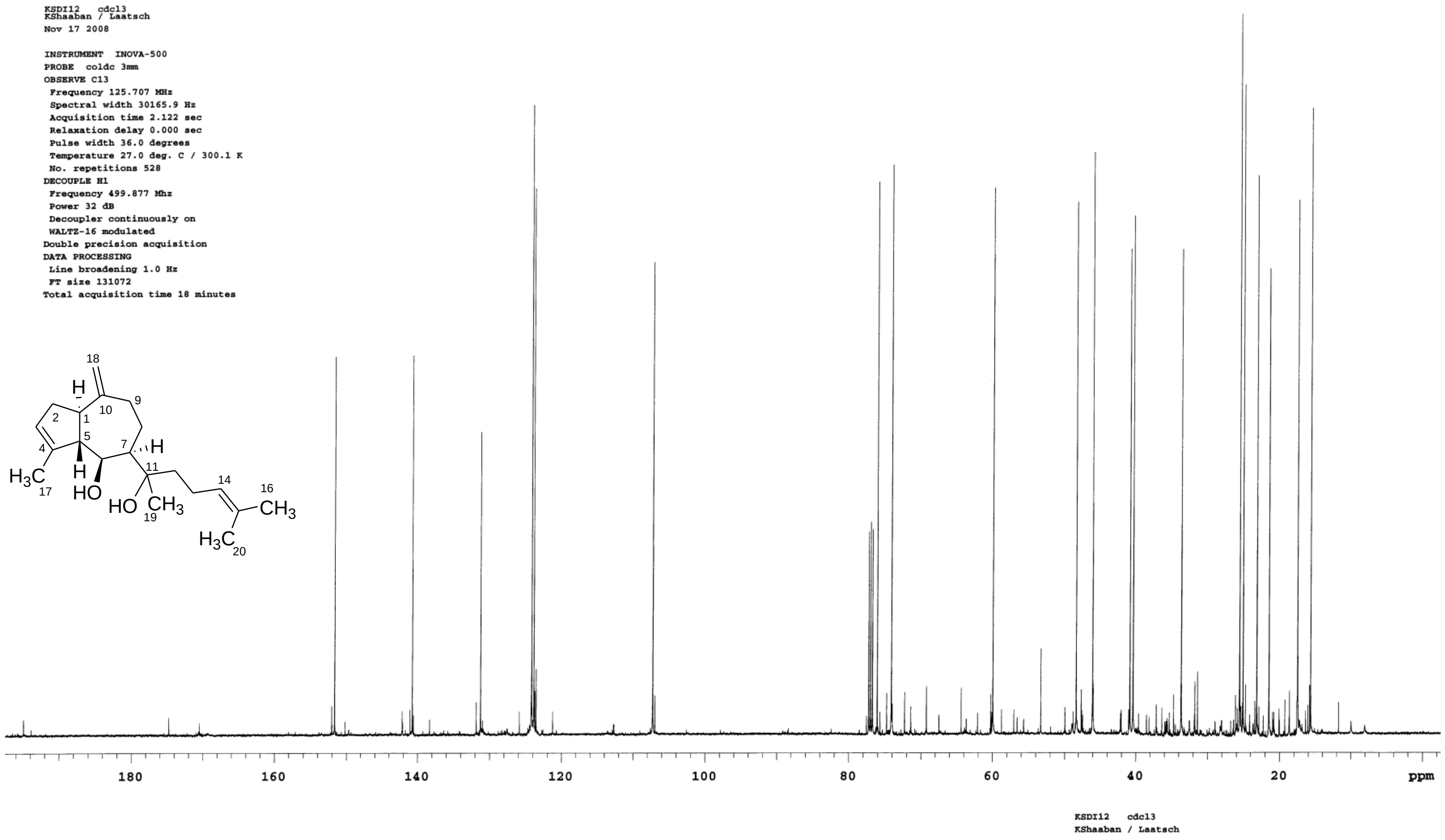

Figure S24. ^{1}H–^{1}H-COSY spectrum ($CDCl_3$, 300 MHz) of dictyol E (**4**).

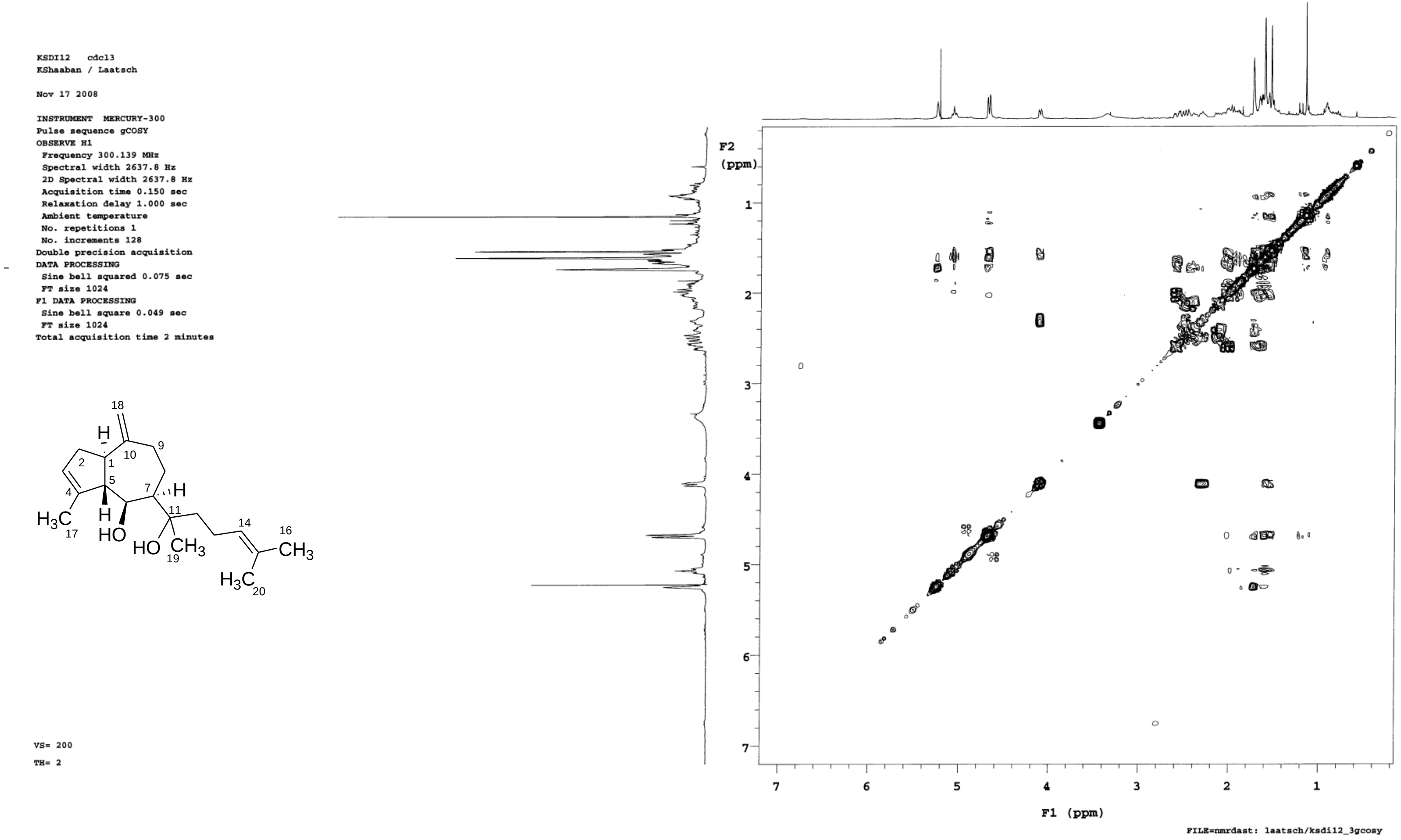

Figure S25. HSQC spectrum ($CDCl_3$, 300 MHz) of dictyol E (**4**).

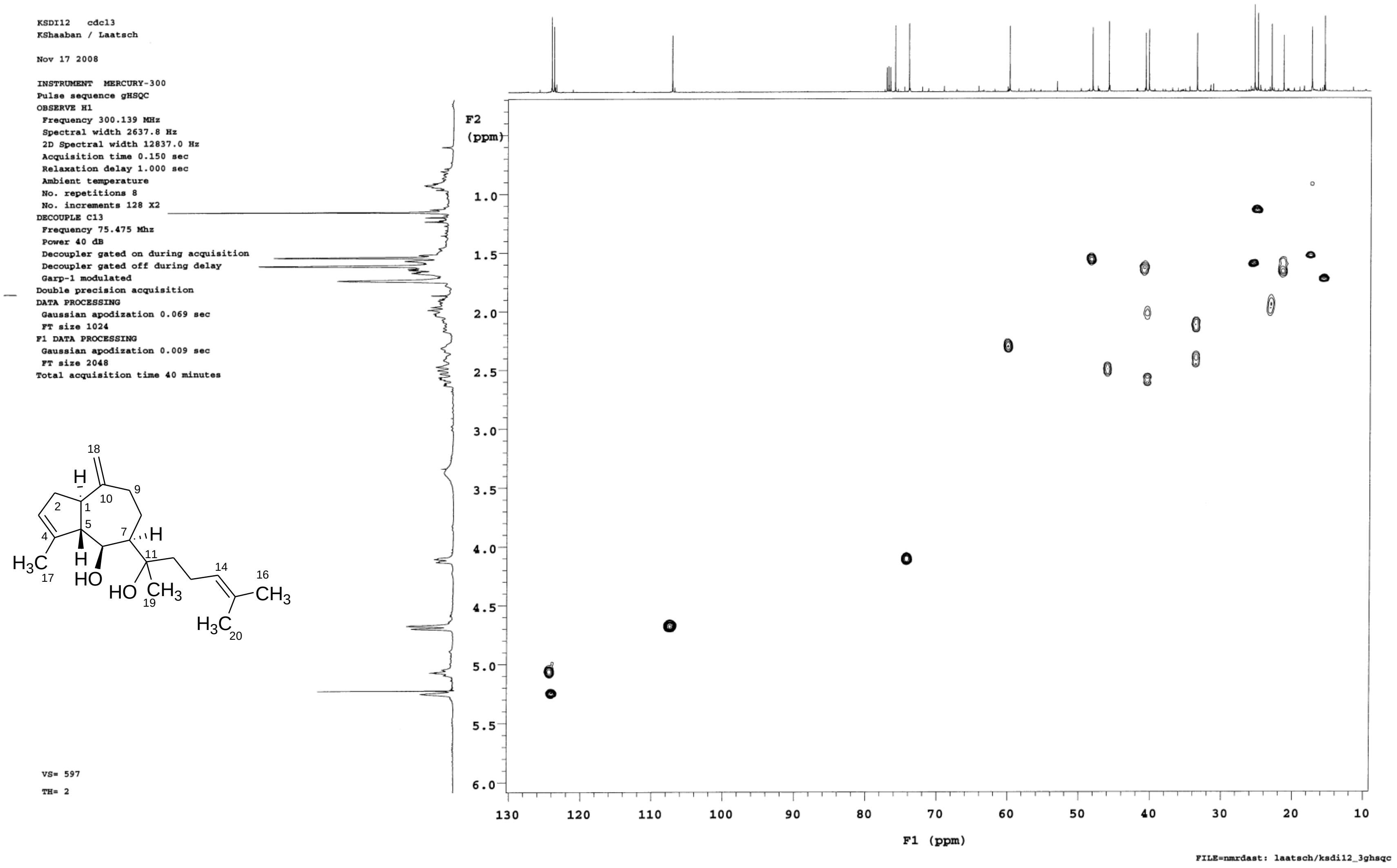

Figure S26. HMBC spectrum ($CDCl_3$, 300 MHz) of dictyol E (**4**).

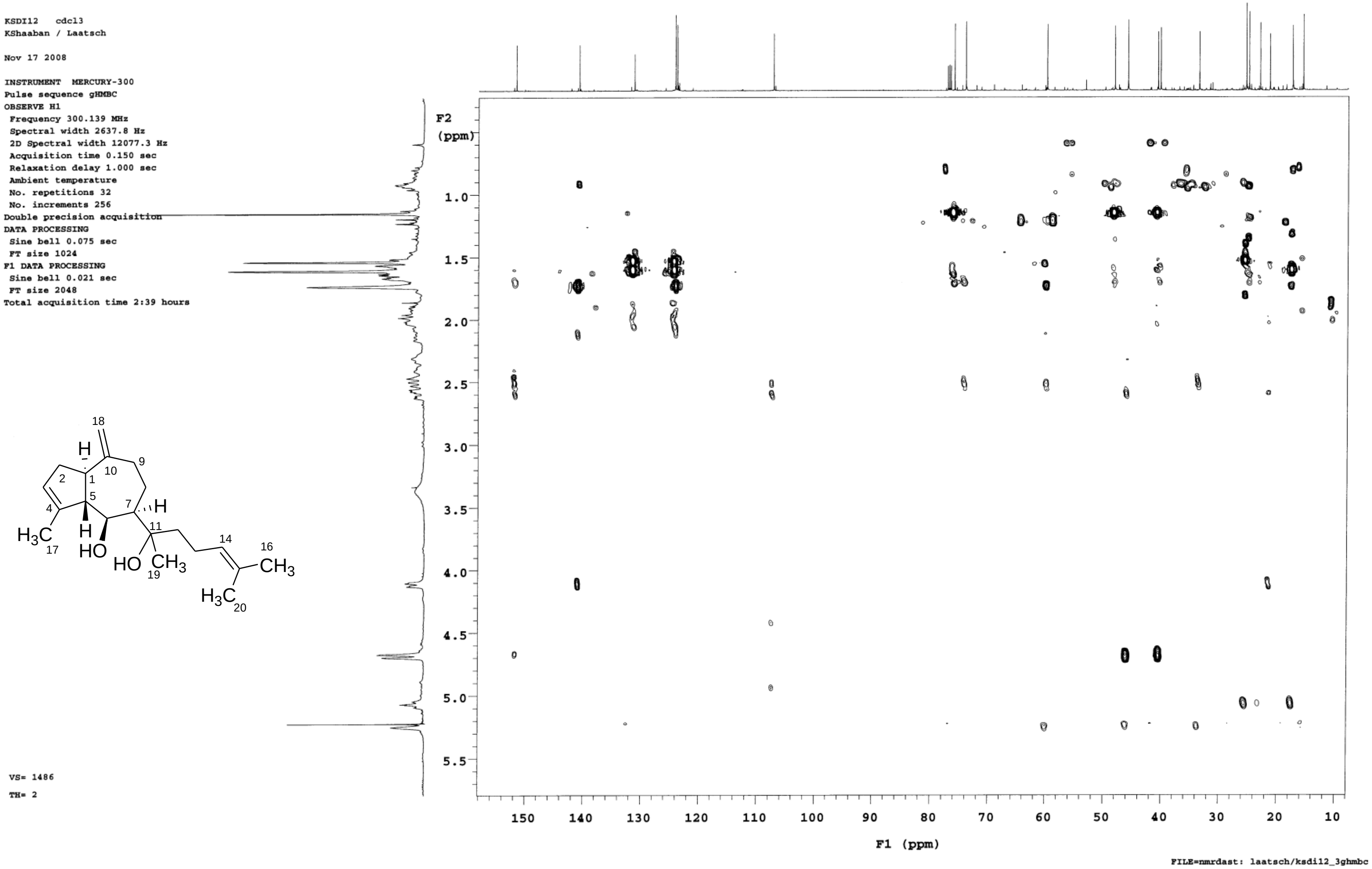

Figure S27. NOESY spectrum ($CDCl_3$, 600 MHz) of dictyol E (**4**).

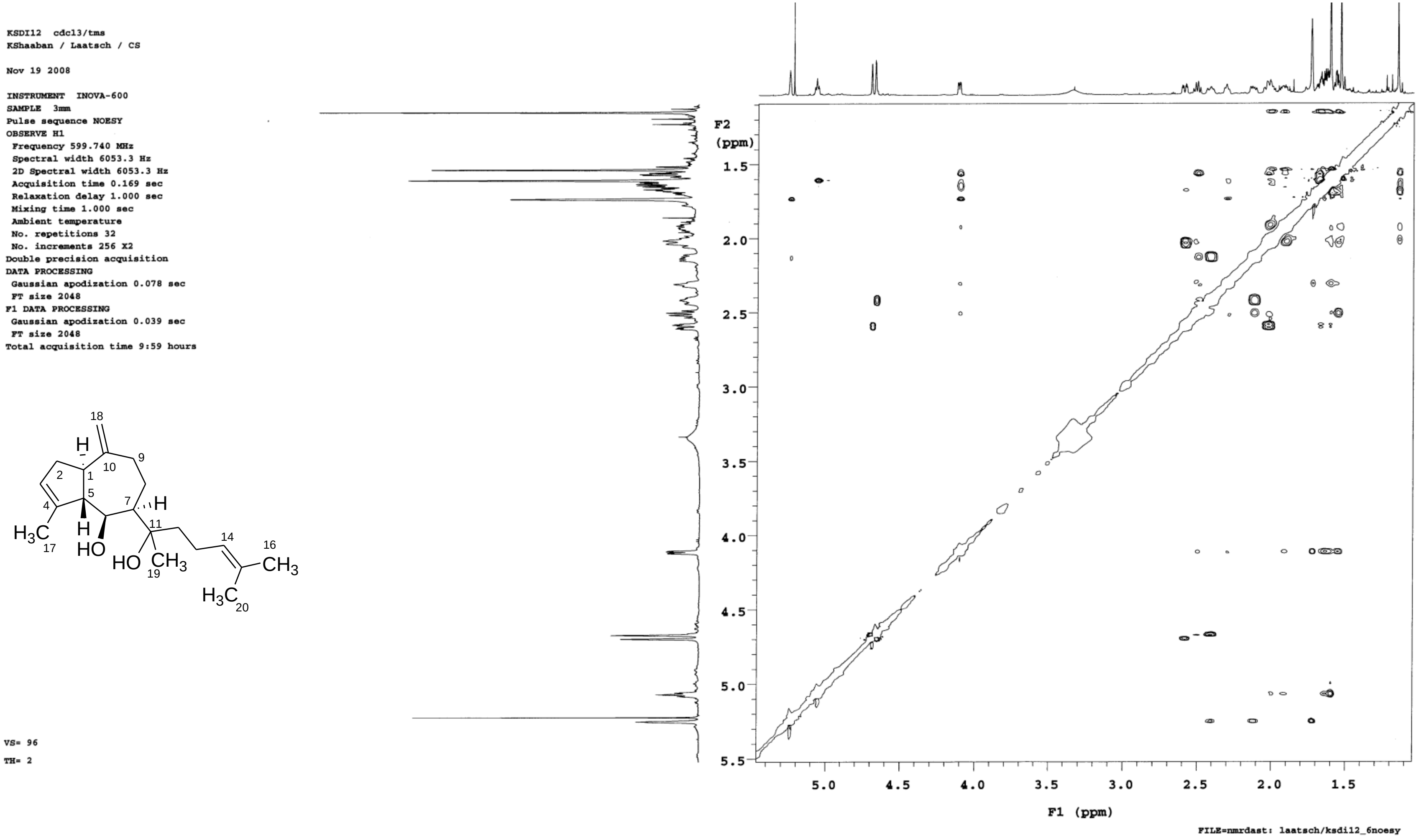

Figure S28. ^{1}H NMR spectrum ($CDCl_3$, 300 MHz) of *cis*-africanan-1α-ol (**5a**).

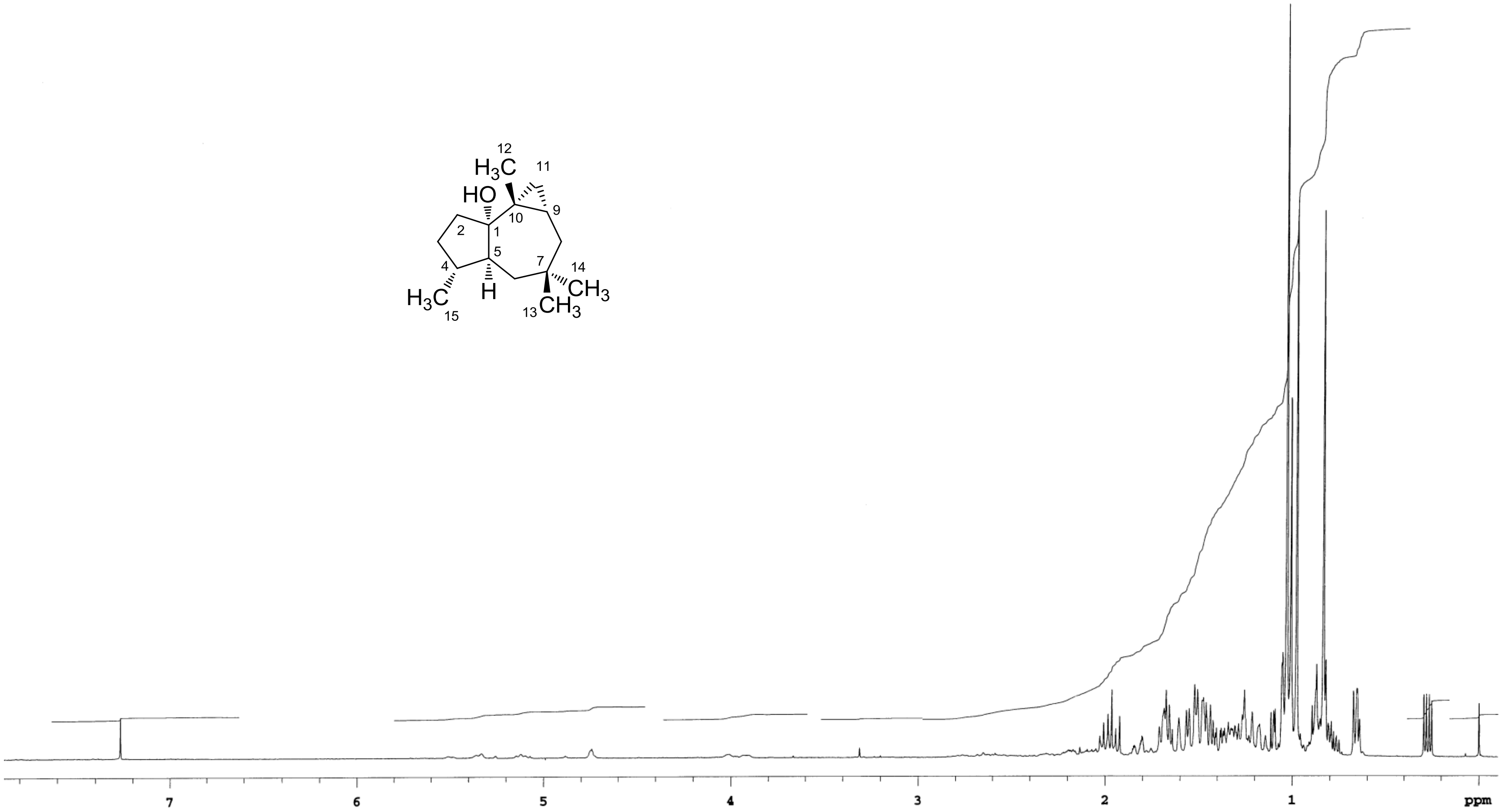

Figure S29. ^{13}C NMR spectrum ($CDCl_3$, 125 MHz) of *cis*-africanan-1α-ol (**5a**).

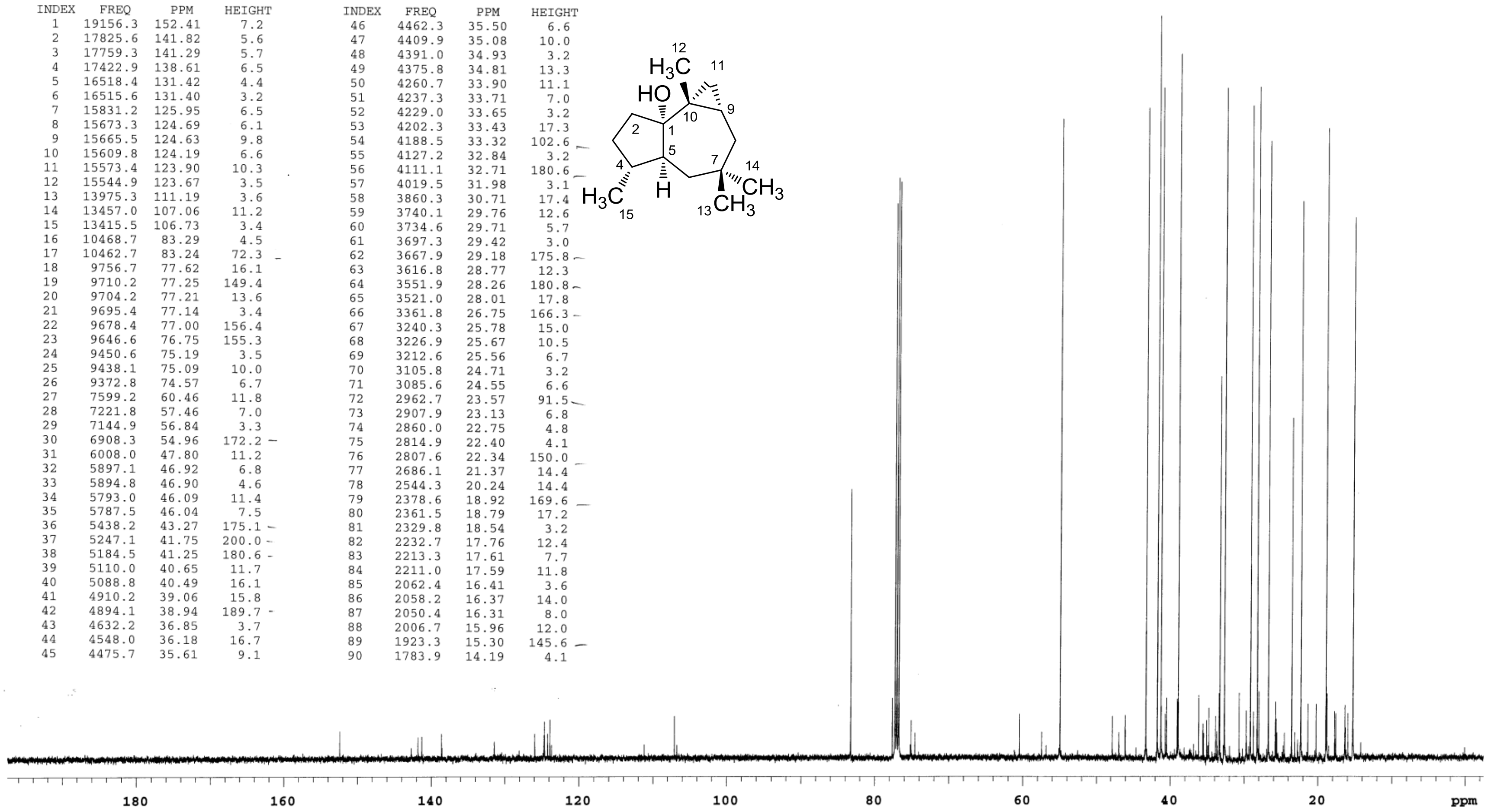

INDEX	FREQ	PPM	HEIGHT
1	19156.3	152.41	7.2
2	17825.6	141.82	5.6
3	17759.3	141.29	5.7
4	17422.9	138.61	6.5
5	16518.4	131.42	4.4
6	16515.6	131.40	3.2
7	15831.2	125.95	6.5
8	15673.3	124.69	6.1
9	15665.5	124.63	9.8
10	15609.8	124.19	6.6
11	15573.4	123.90	10.3
12	15544.9	123.67	3.5
13	13975.3	111.19	3.6
14	13457.0	107.06	11.2
15	13415.5	106.73	3.4
16	10468.7	83.29	4.5
17	10462.7	83.24	72.3
18	9756.7	77.62	16.1
19	9710.2	77.25	149.4
20	9704.2	77.21	13.6
21	9695.4	77.14	3.4
22	9678.4	77.00	156.4
23	9646.6	76.75	155.3
24	9450.6	75.19	3.5
25	9438.1	75.09	10.0
26	9372.8	74.57	6.7
27	7599.2	60.46	11.8
28	7221.8	57.46	7.0
29	7144.9	56.84	3.3
30	6908.3	54.96	172.2
31	6008.0	47.80	11.2
32	5897.1	46.92	6.8
33	5894.8	46.90	4.6
34	5793.0	46.09	11.4
35	5787.5	46.04	7.5
36	5438.2	43.27	175.1
37	5247.1	41.75	200.0
38	5184.5	41.25	180.6
39	5110.0	40.65	11.7
40	5088.8	40.49	16.1
41	4910.2	39.06	15.8
42	4894.1	38.94	189.7
43	4632.2	36.85	3.7
44	4548.0	36.18	16.7
45	4475.7	35.61	9.1
46	4462.3	35.50	6.6
47	4409.9	35.08	10.0
48	4391.0	34.93	3.2
49	4375.8	34.81	13.3
50	4260.7	33.90	11.1
51	4237.3	33.71	7.0
52	4229.0	33.65	3.2
53	4202.3	33.43	17.3
54	4188.5	33.32	102.6
55	4127.2	32.84	3.2
56	4111.1	32.71	180.6
57	4019.5	31.98	3.1
58	3860.3	30.71	17.4
59	3740.1	29.76	12.6
60	3734.6	29.71	5.7
61	3697.3	29.42	3.0
62	3667.9	29.18	175.8
63	3616.8	28.77	12.3
64	3551.9	28.26	180.8
65	3521.0	28.01	17.8
66	3361.8	26.75	166.3
67	3240.3	25.78	15.0
68	3226.9	25.67	10.5
69	3212.6	25.56	6.7
70	3105.8	24.71	3.2
71	3085.6	24.55	6.6
72	2962.7	23.57	91.5
73	2907.9	23.13	6.8
74	2860.0	22.75	4.8
75	2814.9	22.40	4.1
76	2807.6	22.34	150.0
77	2686.1	21.37	14.4
78	2544.3	20.24	14.4
79	2378.6	18.92	169.6
80	2361.5	18.79	17.2
81	2329.8	18.54	3.2
82	2232.7	17.76	12.4
83	2213.3	17.61	7.7
84	2211.0	17.59	11.8
85	2062.4	16.41	3.6
86	2058.2	16.37	14.0
87	2050.4	16.31	8.0
88	2006.7	15.96	12.0
89	1923.3	15.30	145.6
90	1783.9	14.19	4.1

Figure S30. ^{1}H–^{1}H-COSY spectrum ($CDCl_3$, 300 MHz) of *cis*-africanan-1α-ol (**5a**).

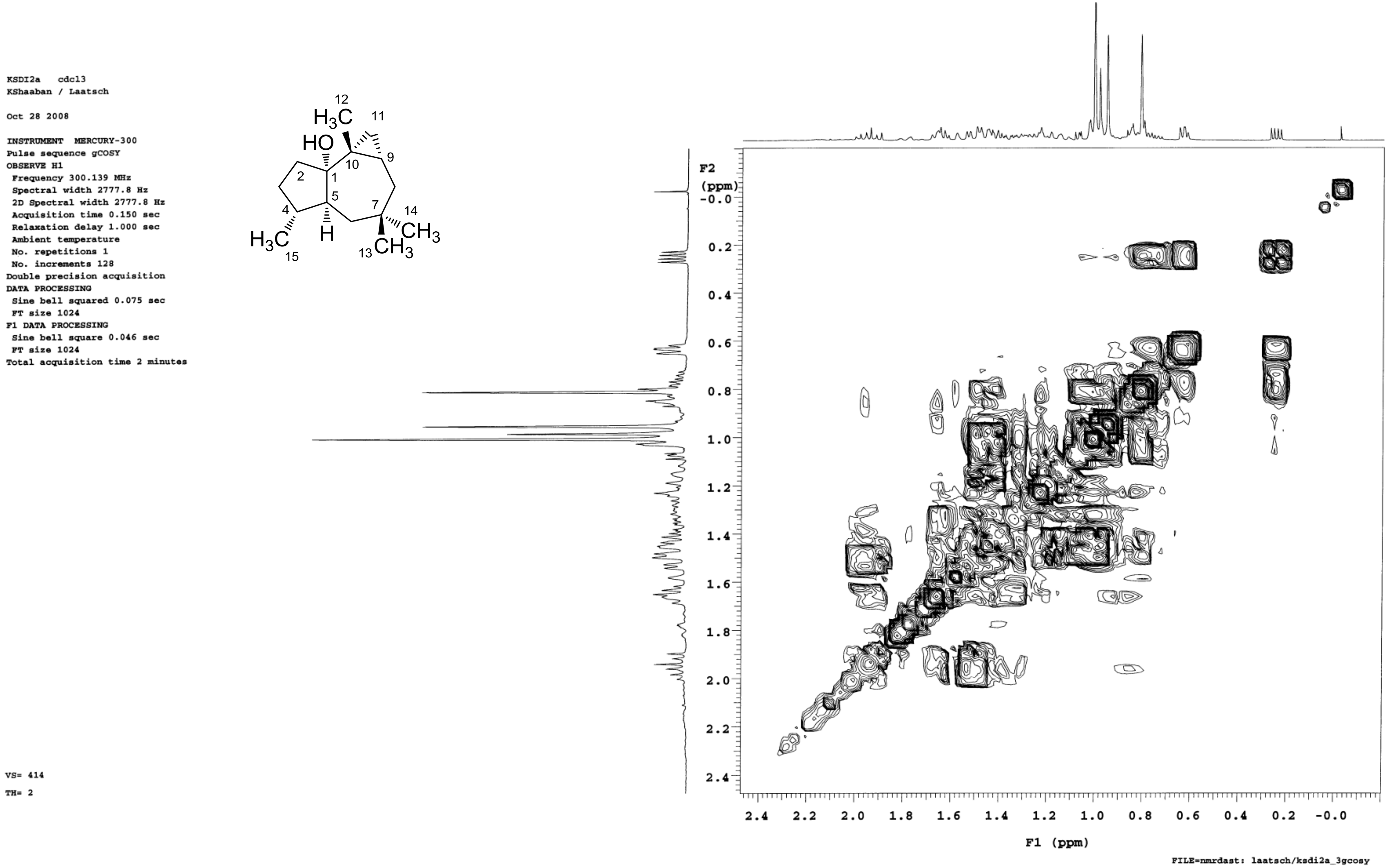

Figure S31. HSQC spectrum ($CDCl_3$, 300 MHz) of *cis*-africanan-1α-ol (**5a**).

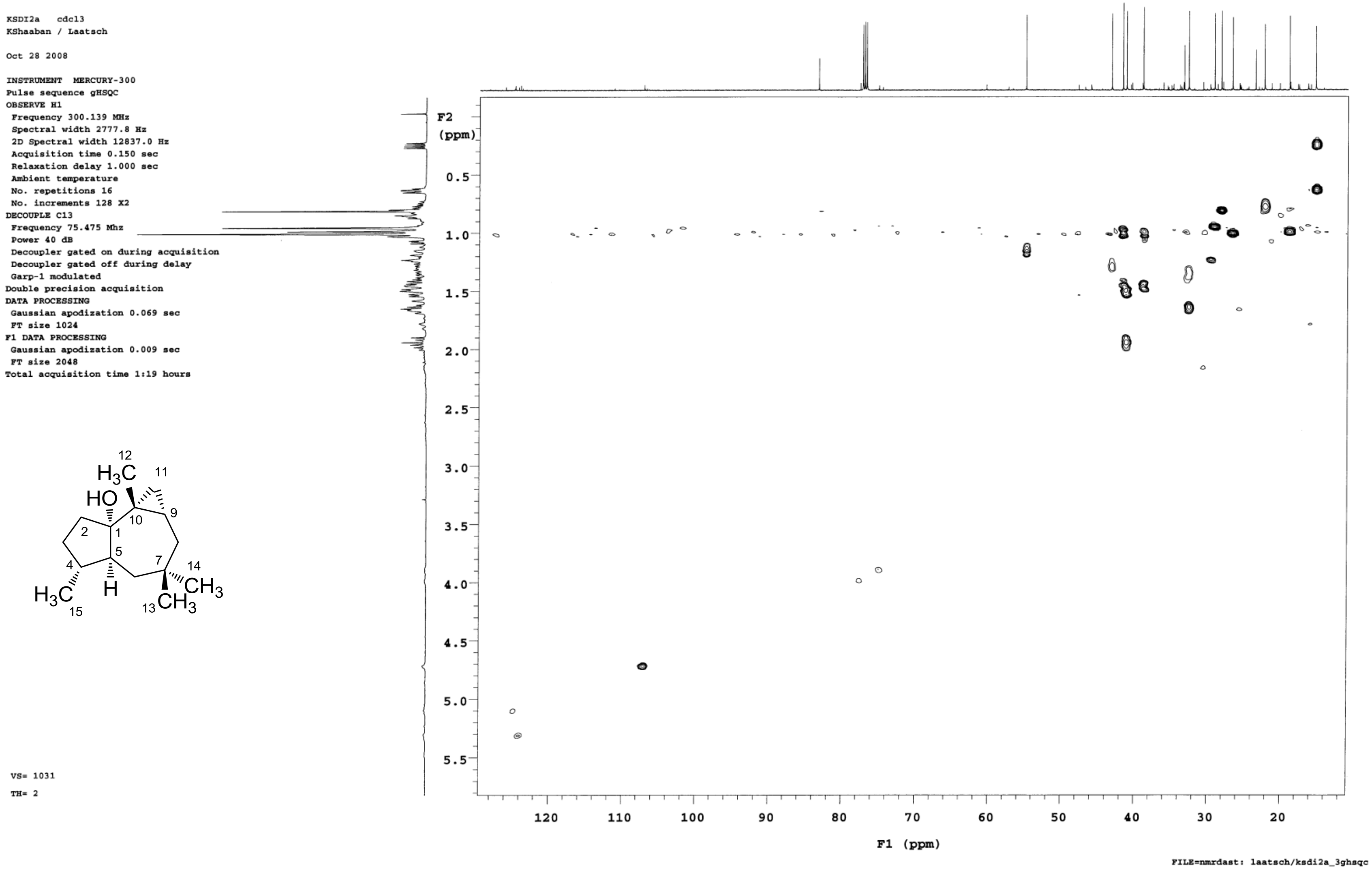

Figure S32. HMBC spectrum ($CDCl_3$, 300 MHz) of *cis*-africanan-1α-ol (**5a**).

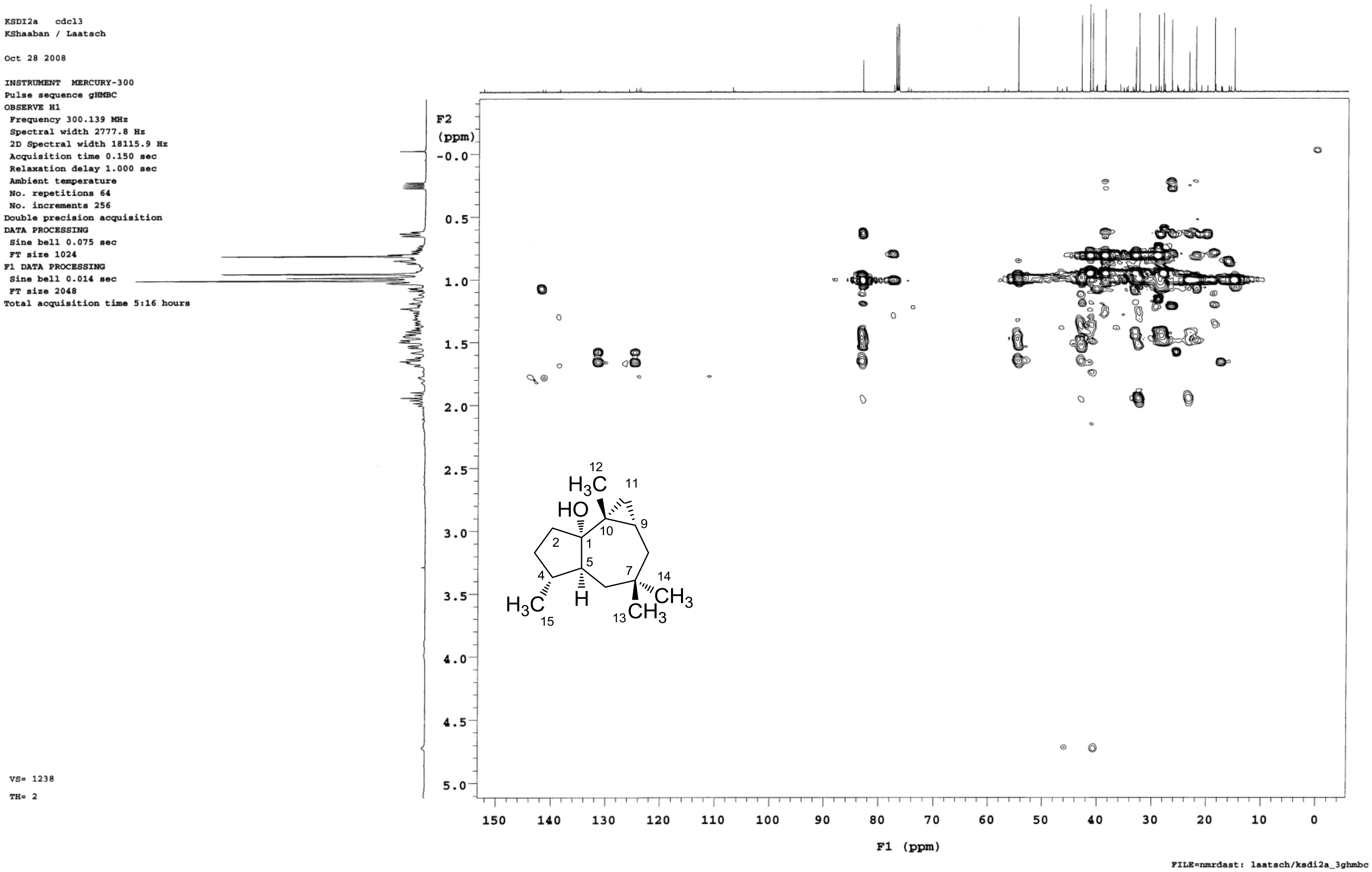

Figure S33. ^{1}H NMR spectrum ($CDCl_3$, 300 MHz) of fucosterol (**6**).

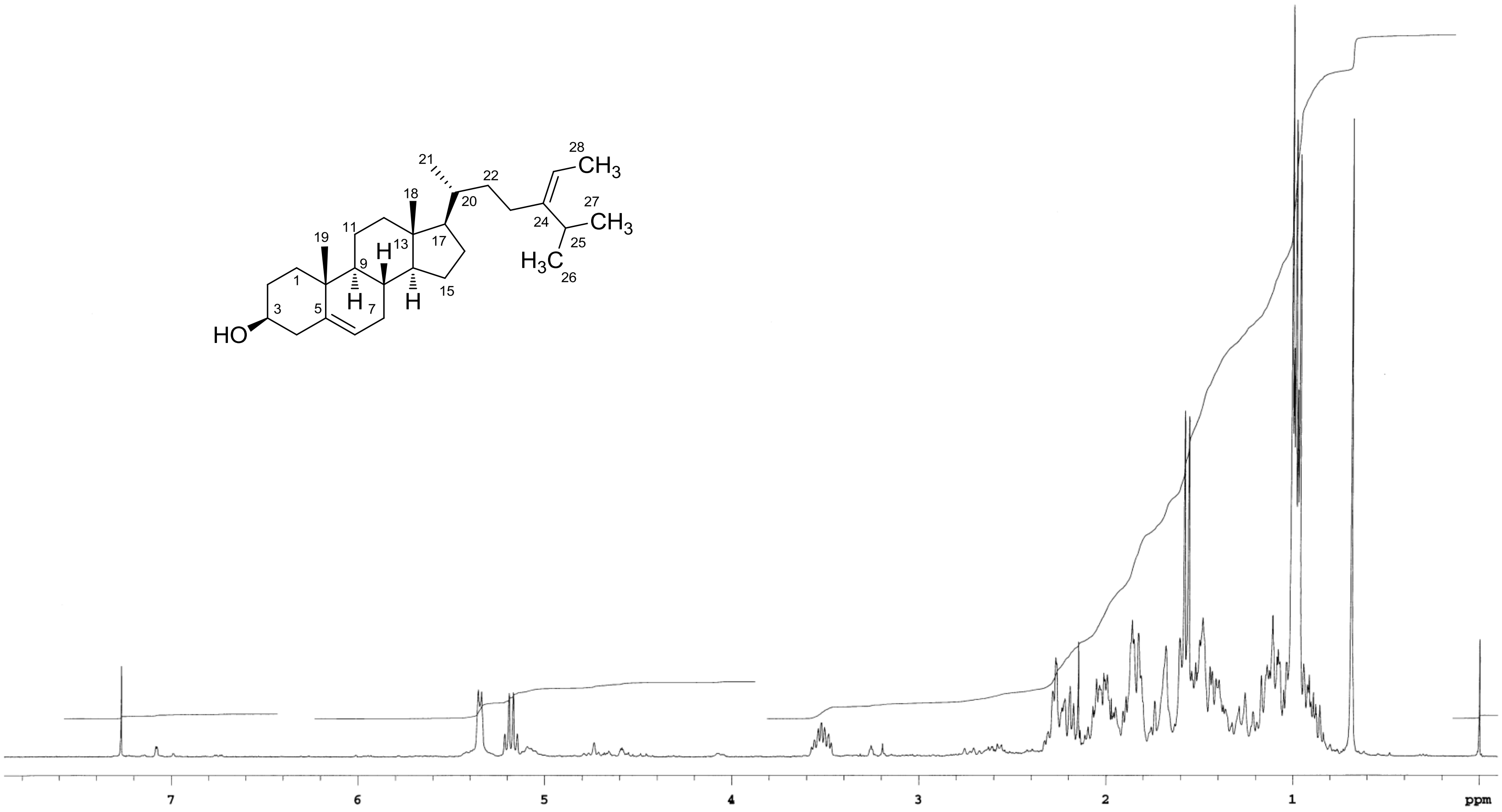

Figure S34. ^{13}C NMR spectrum ($CDCl_3$, 125 MHz) of fucosterol (**6**).

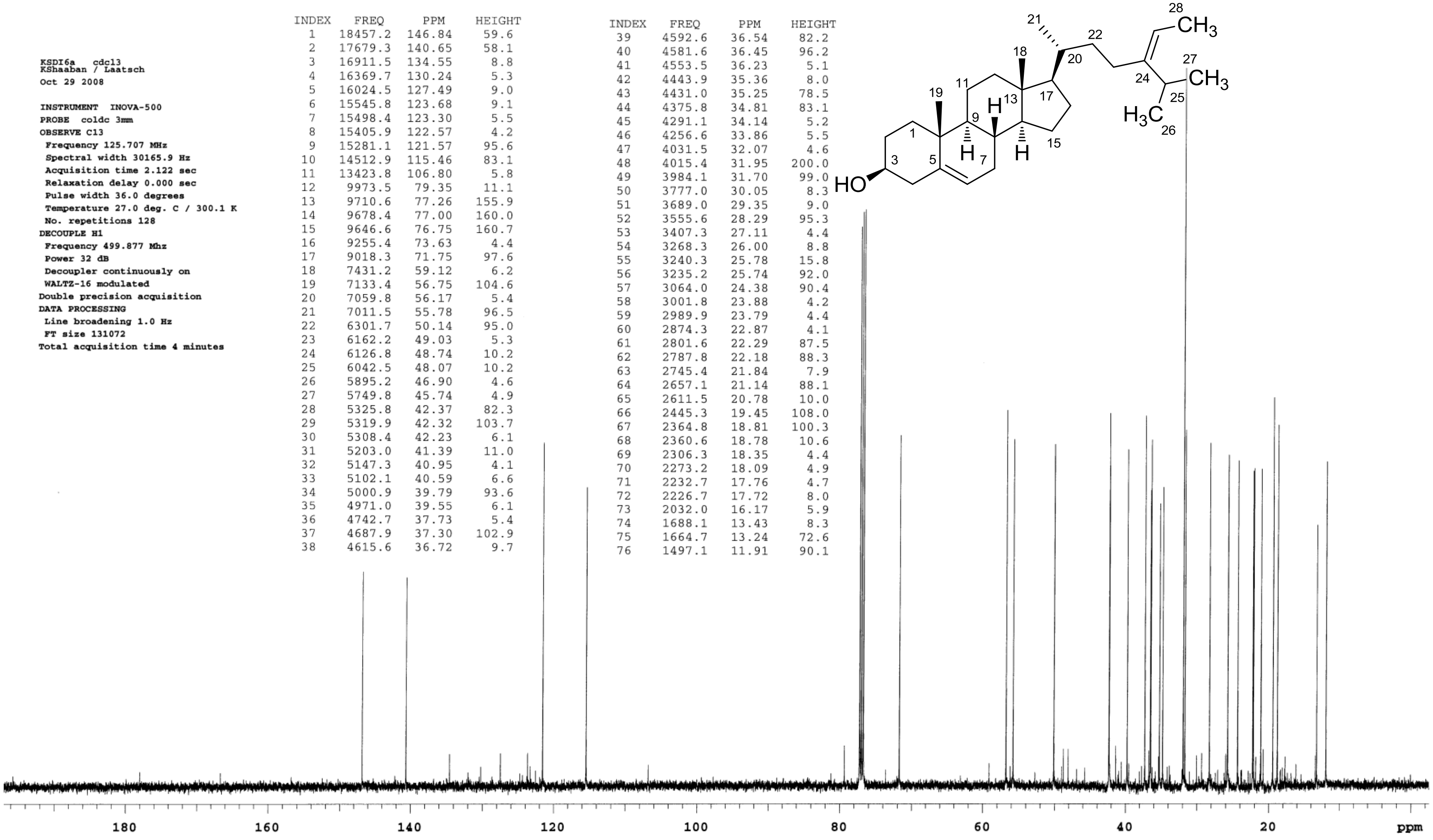

INDEX	FREQ	PPM	HEIGHT
1	18457.2	146.84	59.6
2	17679.3	140.65	58.1
3	16911.5	134.55	8.8
4	16369.7	130.24	5.3
5	16024.5	127.49	9.0
6	15545.8	123.68	9.1
7	15498.4	123.30	5.5
8	15405.9	122.57	4.2
9	15281.1	121.57	95.6
10	14512.9	115.46	83.1
11	13423.8	106.80	5.8
12	9973.5	79.35	11.1
13	9710.6	77.26	155.9
14	9678.4	77.00	160.0
15	9646.6	76.75	160.7
16	9255.4	73.63	4.4
17	9018.3	71.75	97.6
18	7431.2	59.12	6.2
19	7133.4	56.75	104.6
20	7059.8	56.17	5.4
21	7011.5	55.78	96.5
22	6301.7	50.14	95.0
23	6162.2	49.03	5.3
24	6126.8	48.74	10.2
25	6042.5	48.07	10.2
26	5895.2	46.90	4.6
27	5749.8	45.74	4.9
28	5325.8	42.37	82.3
29	5319.9	42.32	103.7
30	5308.4	42.23	6.1
31	5203.0	41.39	11.0
32	5147.3	40.95	4.1
33	5102.1	40.59	6.6
34	5000.9	39.79	93.6
35	4971.0	39.55	6.1
36	4742.7	37.73	5.4
37	4687.9	37.30	102.9
38	4615.6	36.72	9.7

INDEX	FREQ	PPM	HEIGHT
39	4592.6	36.54	82.2
40	4581.6	36.45	96.2
41	4553.5	36.23	5.1
42	4443.9	35.36	8.0
43	4431.0	35.25	78.5
44	4375.8	34.81	83.1
45	4291.1	34.14	5.2
46	4256.6	33.86	5.5
47	4031.5	32.07	4.6
48	4015.4	31.95	200.0
49	3984.1	31.70	99.0
50	3777.0	30.05	8.3
51	3689.0	29.35	9.0
52	3555.6	28.29	95.3
53	3407.3	27.11	4.4
54	3268.3	26.00	8.8
55	3240.3	25.78	15.8
56	3235.2	25.74	92.0
57	3064.0	24.38	90.4
58	3001.8	23.88	4.2
59	2989.9	23.79	4.4
60	2874.3	22.87	4.1
61	2801.6	22.29	87.5
62	2787.8	22.18	88.3
63	2745.4	21.84	7.9
64	2657.1	21.14	88.1
65	2611.5	20.78	10.0
66	2445.3	19.45	108.0
67	2364.8	18.81	100.3
68	2360.6	18.78	10.6
69	2306.3	18.35	4.4
70	2273.2	18.09	4.9
71	2232.7	17.76	4.7
72	2226.7	17.72	8.0
73	2032.0	16.17	5.9
74	1688.1	13.43	8.3
75	1664.7	13.24	72.6
76	1497.1	11.91	90.1

Table S1. GC-MS analysis of the nonpolar fraction I.

Name	R_t (min)	Formula	Mol.Wt.
Nonanal	9.35	$C_9H_{18}O$	142
Tetrahydrothiophen-1,1-dioxide	11.05	$C_4H_8O_2S$	120
β-Bourbonene	13.58	$C_{15}H_{24}$	204
Pentadecane	14.84	$C_{15}H_{32}$	212
Hexadecane	14.86	$C_{16}H_{34}$	226
Heptadecane	17.21	$C_{17}H_{36}$	240
Tetradecanoic acid	17.86	$C_{14}H_{28}O_2$	228
Hexadecanoic acid	19.96	$C_{16}H_{32}O_2$	256
(9*Z*)-Octadecenoic acid methyl ester	21.27	$C_{19}H_{36}O_2$	296
(9*Z*)-Octadecenoic acid	21.60	$C_{18}H_{34}O_2$	282
Diisooctyl adipate	23.85	$C_{22}H_{42}O_4$	370

Table S2. GC-MS analysis of the unsaponifiable part of the petroleum ether extract of *D. dichotoma*.

Name	R_t (min)	Formula	Mol.Wt.
2-Ethyl-1-hexanol	7.09	$C_8H_{18}O$	130
2,2,6,7-Tetramethyl-10-oxatricyclo[4.3.0.1(1,7)decan-5-one (**7**)	12.15	$C_{13}H_{20}O_2$	208
1-Bromo-(4-bromomethyl)-decane	12.43	$C_{11}H_{22}Br_2$	314
N-(4-Bromo-*n*-butyl)-piperidin-2-one (**8**)	13.37	$C_9H_{16}BrNO$	234
tert-Hexadecanethiol (**9**)	14.1	$C_{16}H_{34}S$	258
17-Pentatriacontene	16.1	$C_{35}H_{70}$	490
1-Hexacosene	18.7	$C_{26}H_{52}$	364
Tetrapentacontane	19.75	$C_{54}H_{110}$	758
